# Different osteoarthritis progression rates within early-stage knee osteoarthritis: a retrospective cohort study using machine learning cluster analysis from the osteoarthritis initiative database

**DOI:** 10.1186/s43019-026-00355-z

**Published:** 2026-11-02

**Authors:** Ji Woo Song, Sung Eun Kim, Du Hyun Ro

**Affiliations:** 1 College of Medicine, Yonsei University, Seoul, Republic of Korea; 2 Department of Orthopedic Surgery, Seoul National University Hospital, Seoul, Republic of Korea; 3 Department of Biomedical Informatics, Harvard Medical School, Boston, United States; 4 CONNECTEVE Co., Ltd., Seoul, Republic of Korea

**Keywords:** Knee osteoarthritis, Cluster analysis, Progression rate, Radiographic phenotypes

## Abstract

**Background:**

Knee osteoarthritis (OA) is a heterogeneous disease with varying progression rates among patients within the same severity grade. This study aimed to identify clinically interpretable radiographic phenotypes among knees with baseline Kellgren and Lawrence (KL) grade 2 osteoarthritis and to compare their 4-year radiographic progression patterns using baseline radiographic features, lifetime exercise patterns, and demographic characteristics.

**Methods:**

Data of patients with baseline KL grade 2 from the Osteoarthritis Initiative database were retrieved. Unsupervised clustering was performed using uniform manifold approximation and projection (UMAP) for dimensionality reduction, followed by hierarchical density-based spatial clustering of applications with noise (HDBSCAN) to identify distinct subgroups. The proportion of knees progressing to KL grade ≥ 3 within 4 years was compared between subgroups, followed by a survival analysis until knee replacement or progression to KL grade 4.

**Results:**

A total of 1997 knees with a mean follow-up of 103.3 months (maximum, 108 months) were included. A total of seven subgroups were identified and classified into accelerated osteoarthritis progression (AOP) group and slow osteoarthritis progression (SOP) group according to whether their 4-year KL grade progression proportions were above or below the training-set average. In a supportive long-term survival analysis, the AOP group also showed significantly earlier progression to knee replacement or KL grade 4 than the SOP group (training set, *P* < 0.001; test set, *P* = 0.049), although the absolute difference in mean survival time was modest (8.3 versus 8.5 years in the training set; 8.4 versus 8.6 years in the test set). The AOP group was characterized mainly by lateral joint space narrowing (JSN) and medial JSN with subchondral sclerosis, whereas the SOP group was characterized by medial JSN without subchondral sclerosis, osteophyte-dominant patterns without JSN, or chondrocalcinosis-dominant patterns; these differences were statistically significant across clusters.

**Conclusions:**

Within baseline KL grade 2 knees, clinically interpretable radiographic phenotypes, including lateral JSN, medial JSN with subchondral sclerosis, osteophyte-dominant changes without JSN, and chondrocalcinosis-dominant patterns, were associated with different 4-year KL grade progression rates. These findings support viewing KL grade 2 knee OA as a heterogeneous radiographic stage that may be further stratified into phenotype-based subgroups with different short-term radiographic prognoses.

**Supplementary information:**

The online version contains supplementary material available at https://doi.org/10.1186/s43019-026-00355-z.

## Background

Knee osteoarthritis (OA) is a degenerative disease characterized by pain, stiffness, reduced mobility, and muscle weakness. Its progression and manifestation vary among patients, from asymptomatic to severe, highlighting the heterogeneity of the disease [1].

One widely used method for assessing OA severity is the Kellgren and Lawrence (KL) grading system, which summarizes radiographic OA severity primarily according to osteophyte formation and joint space narrowing [2]. KL grade 2 represents an early-to-intermediate radiographic stage of knee OA, in which conservative management and prognostic monitoring are clinically relevant [3, 4]. Progression from KL grade 2 to KL grade 3 or 4 is clinically important because it reflects advancement to more definite or severe structural OA. However, because KL grade is a global ordinal category, it may obscure more granular structural patterns among knees assigned the same grade. Knees assigned KL grade 2 may still show clinically meaningful radiographic heterogeneity that could be associated with different progression patterns. More detailed phenotyping approaches, including semiquantitative radiographic assessment using the Osteoarthritis Research Society International (OARSI) atlas [5], can characterize individual structural features, such as joint space narrowing, subchondral sclerosis, osteophytes, and chondrocalcinosis, in greater detail.

Previous studies have separately described clinically relevant structural patterns, including isolated lateral versus medial compartment OA [6], subchondral bone remodeling [7], osteophyte-dominant KL grade 2 knees without joint space narrowing [8], and chondrocalcinosis-associated OA [9]. However, it remains unclear whether these different radiographic patterns coexist as distinct subgroups within a single cohort of knees with baseline KL grade 2. Few studies have used an integrative, data-driven approach to identify such subgroups simultaneously by combining detailed radiographic features with demographic characteristics and lifetime exercise exposure.

This gap supports the use of an unsupervised clustering approach, which can group knees according to the similarity of multiple baseline variables without prespecifying outcome labels. By integrating OARSI atlas-based radiographic features, lifetime exercise variables, and demographic characteristics, clustering can explore whether these variables form recognizable multivariable phenotypes within baseline KL grade 2 knees. Accordingly, this study aimed to identify clinically interpretable phenotypic subgroups among knees with baseline KL grade 2 osteoarthritis using baseline radiographic features, lifetime exercise patterns, and demographic characteristics, and to compare their 4-year radiographic progression patterns.

## Methods

### Study population

Patient data were obtained from the Osteoarthritis Initiative (OAI), a multicenter, longitudinal, prospective observational cohort study of knee osteoarthritis that enrolled 4796 participants aged 45 to 79 years at four US clinical centers (Baltimore, Maryland; Columbus, Ohio; Pittsburgh, Pennsylvania; and Pawtucket, Rhode Island) [5]. Participants underwent protocol-driven follow-up assessments at scheduled visits rather than returning only when seeking treatment, including in-clinic visits at baseline, 12, 24, 36, 48, 72, and 96 months. The analysis in the present study was knee-specific, treating each knee independently. Eligible knees were those with baseline KL grade 2 in the OAI central reading dataset and available 48-month centrally read KL grade data to define 4-year radiographic progression. Knees were also required to have baseline demographic variables, lifetime exercise variables, and baseline radiographic variables used for clustering; knees with missing body mass index (BMI) values were excluded before the final analysis. Exercise-related variables were derived from the baseline OAI clinical questionnaire and reflected retrospectively reported lifetime physical activity across predefined age intervals [10]. As this database is openly accessible, approval from the Institutional Review Board (IRB) was not required.

### Input variables and preprocessing

Demographic factors, including age, sex, BMI, radiographic variables, and the amount of exercise were collected.

Radiographic variables were obtained from the OAI central reading semiquantitative knee radiograph dataset (Boston University). All patients had radiographic evaluation data (OARSI atlas) at baseline, including KL grade and 18 features: osteophyte grades 0–3; subchondral sclerosis grades 0–3; subchondral cysts grades 0–1 for medial/lateral femur/tibia; JSN grades 0–3; chondrocalcinosis grades 0–1 for medial/lateral compartments; bone attrition grades 0–3 for medial/lateral tibia. For radiographic variables based on the OARSI atlas, missing values were present in 178 of 1997 knees (8.9%). Before imputation, the observed distributions of the affected ordinal radiographic variables were zero-dominant: grade 0 accounted for 84.6% of observed subchondral sclerosis feature values, 98.8% of observed subchondral cyst feature values, and 99.97% of observed attrition feature values. Accordingly, the observed median and the resulting imputed value were 0 for each affected variable. Grades of bone attrition and subchondral cysts were used in clustering analysis but not shown in the results due to extremely low prevalence (eight cases of medial tibia cysts, one case of lateral femur cyst, one case of grade one lateral attrition, and no other cases of cyst/attrition) and lack of statistical significance.

Exercise data were included to quantify lifetime exercise load. From the 93 exercise-related variables in the OAI database, we simplified the dataset by selecting exercise frequency and type, calculating the total number of lifetime exercise sessions. This was done by identifying the most frequently performed exercises from a list of 37 types. With reference to the loading concepts described by Buckwalter [11] and Schirò et al. [12], these exercises were categorized into three groups: low, moderate, and high impact on OA. The cumulative impact was calculated using the formula: (number of years) × (number of months per year) × (number of times per month). Detailed methodology is provided in the Supplementary Methods.

### Outcome measures

The progression of knee OA was defined as reaching a KL grade of 3 or 4 in radiographic evaluations after 4 years. A 4-year follow-up period was selected because 48 months provides both a well-populated OAI time point and a clinically meaningful interval for radiographic progression, as prior studies have commonly used 48-month outcomes to characterize structural worsening and accelerated progression in knee OA [13, 14]. To visualize the course of knee OA, a 9-year survival analysis was conducted, leveraging the maximum follow-up data available. The end point event in the survival analysis was defined as (1) undergoing knee replacement surgery or (2) progression to KL grade 4.

### Model development

Patient data were randomly divided into two groups: a training set (70%) and a test set (30%) using a fixed random seed (random_state = 42). This split was used as an internal reproducibility framework rather than as supervised model training. To classify patients within KL grade 2 into subgroups, an unsupervised clustering method was used [15]. The 24 baseline variables (age, sex, BMI, three lifetime exercise-load measures, and 18 radiographic features) were standardized using the means and standard deviations estimated in the training set, and the same transformation was applied to the test set. Uniform Manifold Approximation and projection (UMAP) was then used to reduce the standardized variables to a two-dimensional representation [16]. UMAP maps the high-dimensional data into a lower-dimensional space to facilitate visualization while preserving its original topological structure. Because HDBSCAN identifies clusters based on local density, this dimensionality-reduction step was also intended to reduce sparsity in the original high-dimensional feature space and to provide a lower-dimensional representation suitable for density-based clustering. This step involved fitting UMAP in the training set to learn the low-dimensional structure of the baseline variables; the held-out test set was then projected using the same UMAP transformation to examine whether comparable phenotypic patterns could be observed in an independent subset.

Hierarchical density-based spatial clustering of applications with noise (HDBSCAN) was then fitted independently to the training- and test-set representations [17]. For reproducibility, the final analysis used test_size = 0.3 and random_state = 42 for the data split, UMAP with n_neighbors = 11, min_dist = 0.04, and random_state = 42, and HDBSCAN with min_cluster_size = 10. Because the test set was smaller than the training set, different min_samples values were used for train (50) and test (25) to avoid disproportionately labeling test samples as noise while preserving comparable density-based cluster detection. Hyperparameters were initially selected through outcome-independent exploratory assessment of cluster separation, the silhouette coefficient, the proportion of observations classified as noise, and cluster-size adequacy; progression and survival outcomes were not considered. Robustness was subsequently assessed in the training set using a full Cartesian grid in which all four hyperparameters were varied jointly rather than one at a time (4050 combinations): UMAP n_neighbors values from 3 to 19 in increments of two and min_dist values of 0.01, 0.02, 0.04, 0.08, and 0.16, together with HDBSCAN min_cluster_size values from 5 to 50 in increments of five and min_samples values from 10 to 90 in increments of ten, with UMAP random_state fixed at 42. Density-based clustering validation (DBCV) and the silhouette coefficient after excluding noise observations were used as intrinsic cluster-quality measures, while cluster count and noise proportion were reported descriptively; adjusted Rand index (ARI), including noise as a label, was used only to quantify post hoc concordance with the primary clustering solution.

Because HDBSCAN was fitted independently in the two sets, the numeric cluster labels had no inherent train–test correspondence. To align the clusters, centroids were calculated from the 24 standardized baseline variables, and cosine similarity was computed for all 49 train–test cluster pairs. The Hungarian algorithm identified the outcome-independent one-to-one assignment maximizing total similarity, after which the test-set clusters were relabeled. Progression and survival outcomes were not used for matching; the matched clusters’ 4-year KL grade progression rates were subsequently compared using Pearson and Spearman correlation coefficients.

### Data analysis

Data analysis was conducted using Python 3.10.12 (Python Software Foundation, DE, USA). The Kruskal–Wallis test was used for ordinal variables (e.g., osteophyte grades and JSN grades) and continuous variables such as age and exercise engagement. The chi-squared test was used for binary categorical variables (e.g., sex, progression from KL grade 2 to 3 or 4). Kaplan–Meier survival curves were used, with Fleming–Harrington weighted log-rank tests for statistical comparisons.

## Results

**Fig. 1:**
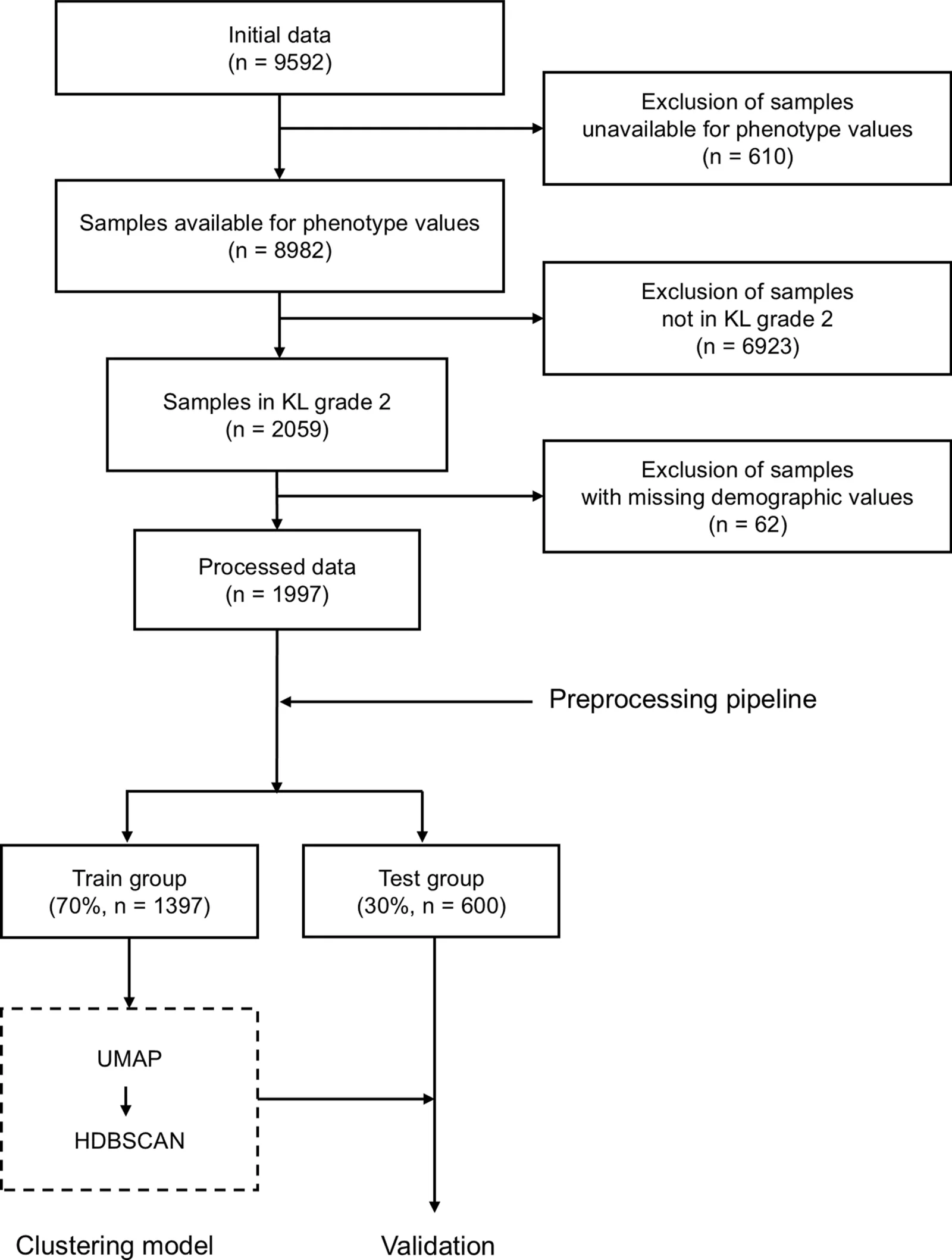
Flow chart of research population. Flow chart illustrates the research population, data preprocessing, exclusions, and partitioning into training and test groups for clustering analysis using UMAP and HDBSCAN. *HDBSCAN* hierarchical density-based spatial clustering of applications with noise, *UMAP* uniform manifold approximation and projection for dimension reduction

Of the 9592 knees in the OAI database, 2558 knees had baseline KL grade 2 in the OAI central reading dataset. After applying the study criteria, including availability of 48-month radiographic progression assessment and baseline variables required for clustering, and after excluding knees with missing BMI values, 1997 knees were included in the final analysis (Fig. 1). The average follow-up period was 103.3 ± 13.7 (mean ± standard deviation) months. Baseline characteristics were generally comparable between the training and test sets, although lateral joint space narrowing was more prevalent in the test set (14% versus 11%; *P* = 0.037; Supplementary Table S-1). The baseline characteristics of the knees which progressed to KL grade 3 or 4 or stayed the same, regardless of subgroups, are detailed in Supplementary Table S-2.

### Subgroup identification and train–test reproducibility

**Fig. 2:**
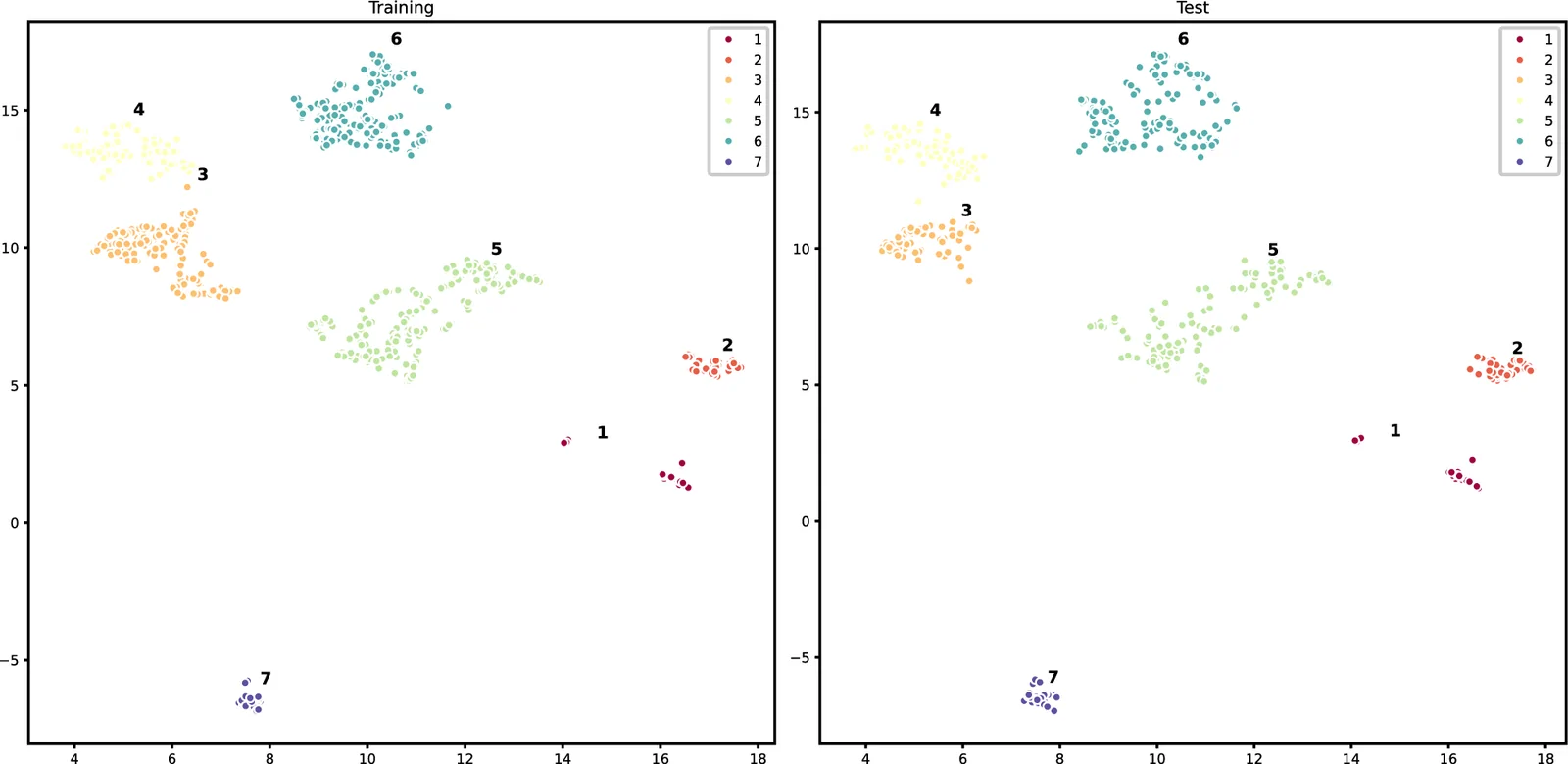
Clustering results using UMAP-enhanced HDBSCAN of **a** the training set and **b** the test set. Both datasets show seven distinct subgroups with broadly comparable phenotypic patterns. *HDBSCAN* hierarchical density based spatial clustering of applications with noise, *UMAP* uniform manifold approximation and projection for dimension reduction

**Fig. 3:**
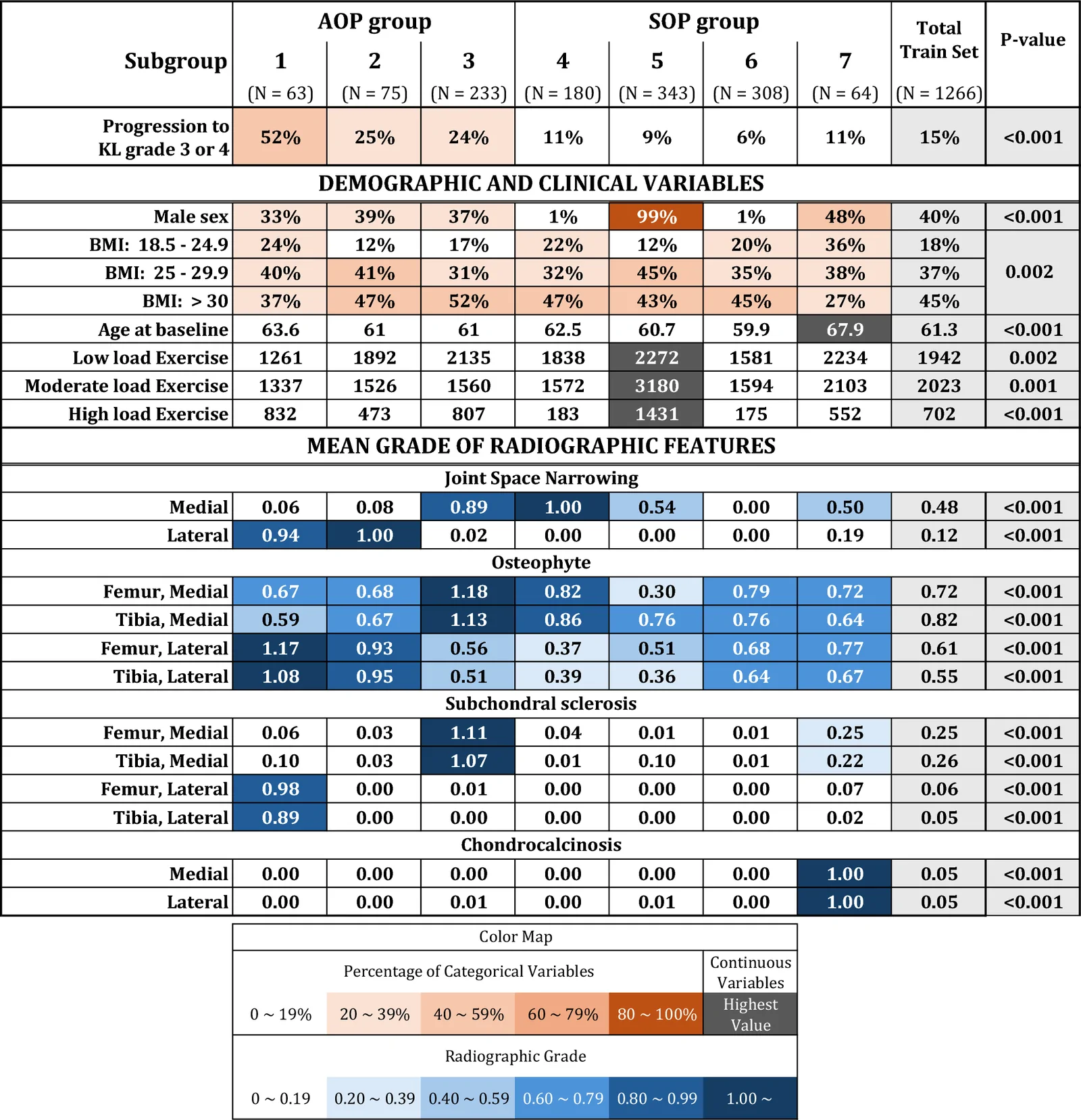
The proportion of knees progressed to KL grade 3 or 4 within 4 years and characteristics of subgroups within the training set depicted via heatmap-style table. For categorical variables, percentage of each subgroup is shown. For continuous variables, the mean value of each subgroup is shown. *AOP group* accelerated osteoarthritis progression group, *SOP group* slow osteoarthritis progression group, *BMI* body mass index, *JSN* joint space narrowing

**Fig. 4:**
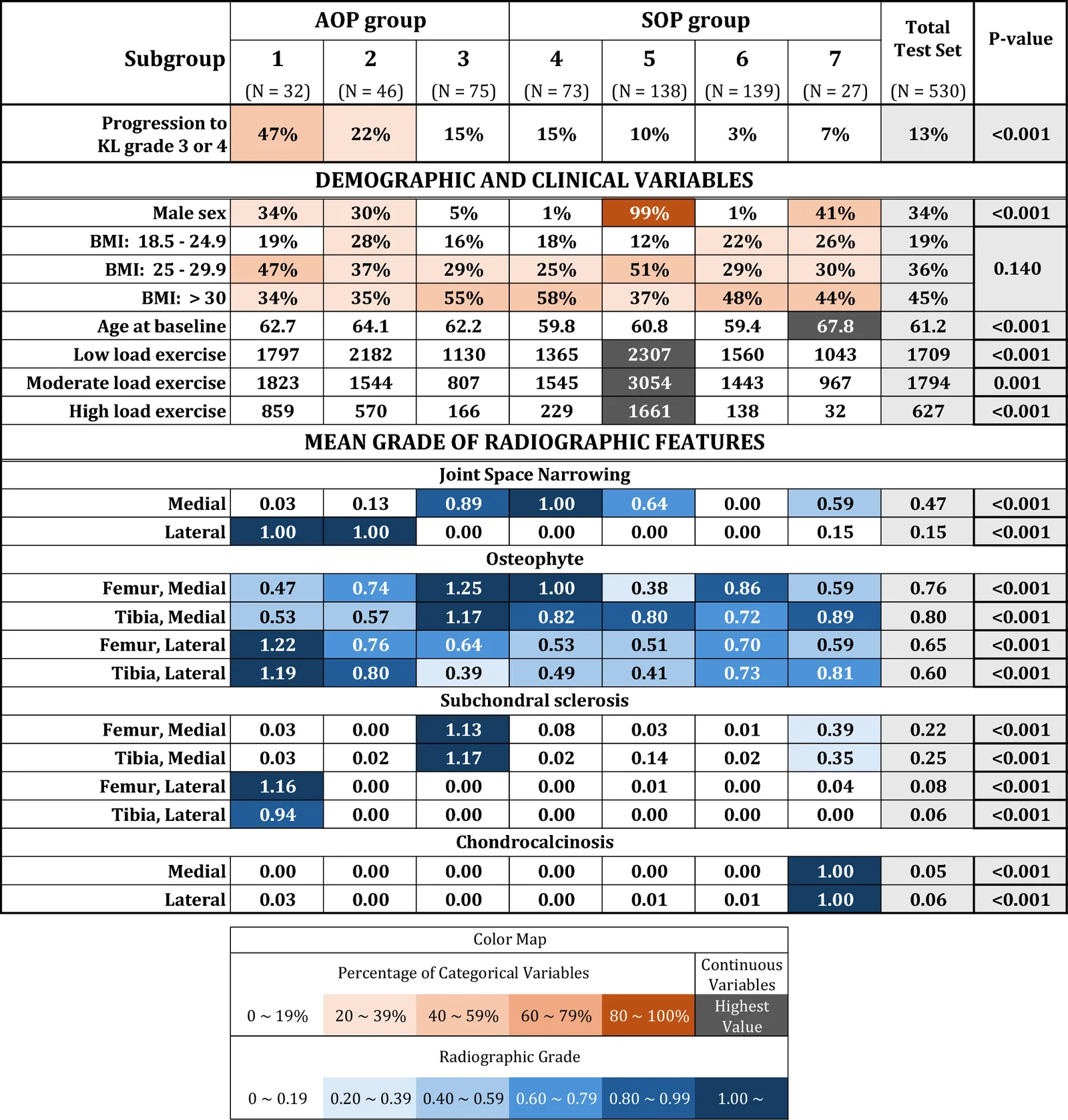
The proportion of knees progressed to KL grade 3 or 4 within 4 years and characteristics of subgroups within the test set depicted via heatmap-style table. For categorical variables, percentage of each subgroup is shown. For continuous variables, the mean value of each subgroup is shown. *AOP group* accelerated osteoarthritis progression group, *SOP group* slow osteoarthritis progression group, *BMI* body mass index, *JSN* joint space narrowing

Figure 2 shows the subgroups from the training and test set via UMAP and HDBSCAN. The training set (Fig. 2a) and test set (Fig. 2b) were both divided into seven subgroups. Baseline characteristics differed significantly across the seven subgroups in both datasets, particularly sex, age, low-, moderate-, and high-load lifetime exercise exposure, and compartment-specific patterns of joint space narrowing, osteophytes, subchondral sclerosis, and chondrocalcinosis; BMI differed significantly in the training set but not in the test set. The proportion of knees progressing to KL grade 3 or 4 within 4 years also differed across the seven subgroups in both datasets (*P* < 0.001), ranging from 52% to 6% in the training set and from 47% to 3% in the test set (Figs. 3 and 4). Among the 201 knees not assigned to a cluster, 43 (21.4%) progressed to KL grade 3 or 4 within 4 years (training set, 26/131 [19.8%]; test set, 17/70 [24.3%]).

Across the full Cartesian grid, 482 of 4050 training-set parameter combinations yielded seven clusters, of which 240 showed high concordance with the primary clustering solution (ARI ≥ 0.90). The primary solution had a DBCV of 0.628 (91st percentile among seven-cluster refits) and a non-noise silhouette coefficient of 0.684, while high-concordance seven-cluster solutions occurred across broad ranges of all four hyperparameters (Supplementary Fig. S-3).

Train–test reproducibility was then assessed using the outcome-independent cluster matching. Each of the seven test-set clusters was paired with its most similar training-set cluster (Supplementary Fig. S4). The mean matched-pair cosine similarity was 0.975 (range, 0.947–0.997) across the 24 standardized baseline variables and 0.986 for the 18 radiographic variables. The matched clusters’ 4-year KL grade progression rates were also highly correlated (Pearson *r* = 0.965; Spearman rho = 0.929).

### Defining accelerated and slow osteoarthritis progression groups

In the training set, the average proportion of progression to KL grade ≥ 3 was 15%. Subgroups 1, 2, and 3 showed higher progression rates than the average, while subgroups 4, 5, 6, and 7 showed lower progression rates. These were designated as the accelerated osteoarthritis progression (AOP) group and the slow osteoarthritis progression (SOP) group, respectively. The progression rates of these groups showed a statistically significant difference (*P* < 0.001).

In the test set, the average proportion of progression to KL grade ≥ 3 was 13%. Subgroups 1, 2, and 3 showed progression rates of 47%, 22%, and 15% respectively, while subgroups 4, 5, 6, and 7 showed progression rates of 15%, 10%, 3%, and 7% respectively. The progression rates of the two groups in the test set also showed a statistically significant difference (*P* < 0.001).

### Distinctive features of AOP and SOP groups

**Fig. 5:**
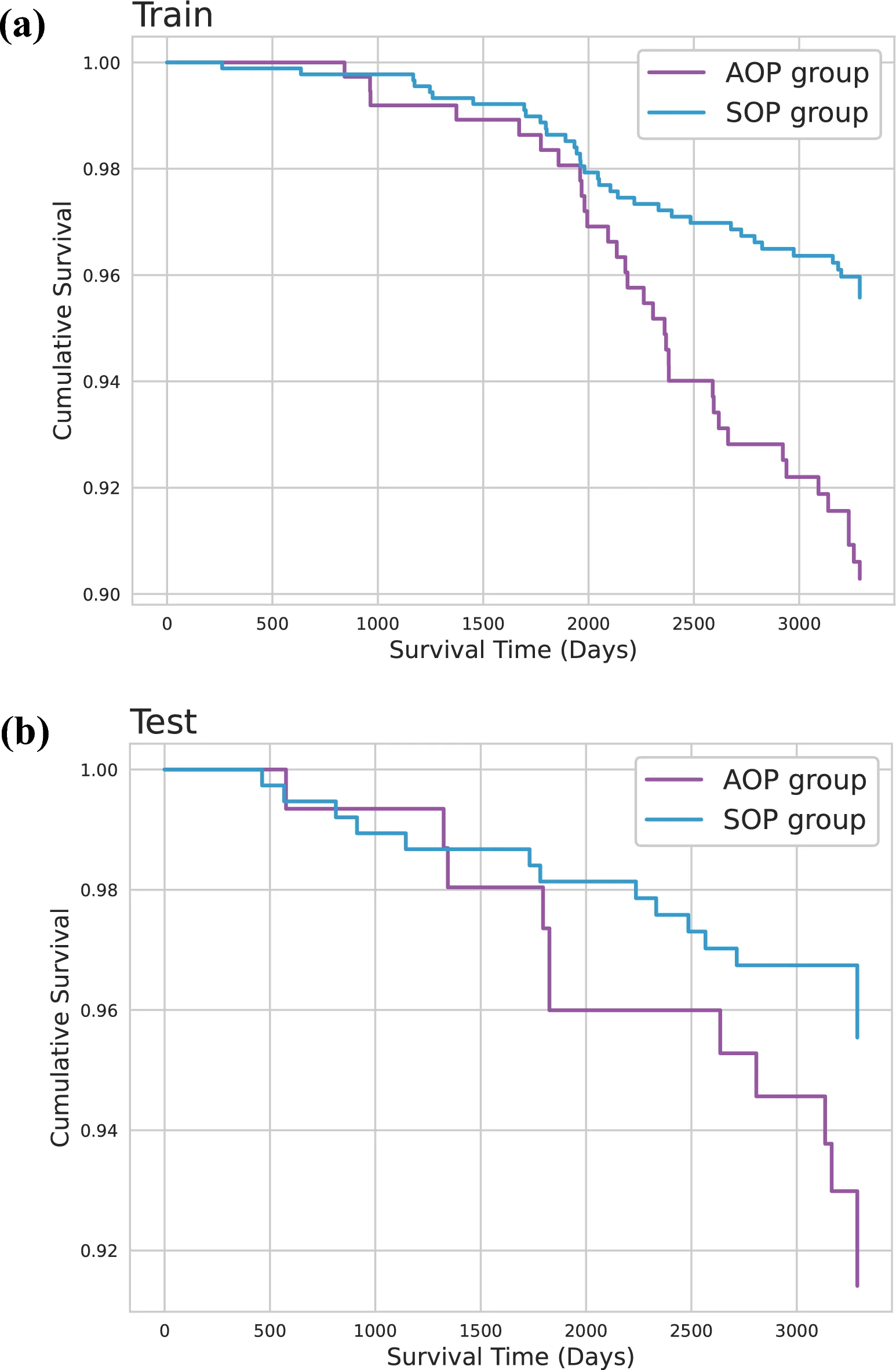
Representative radiographs of six major radiographic phenotype patterns identified among the seven subgroups. **a** Subgroup 1, lateral JSN with subchondral sclerosis; **b** subgroup 2, lateral JSN without subchondral sclerosis; **c** subgroup 3, medial JSN with subchondral sclerosis; **d** subgroups 4 and 5, medial JSN without subchondral sclerosis; **e** subgroup 6, osteophyte-dominant without JSN; **f** subgroup 7, chondrocalcinosis; *JSN* joint space narrowing

Figure 5 shows representative X-ray images of patients in the AOP and SOP groups from the OAI database. Detailed characteristics of the AOP and SOP groups are provided in Fig. 3 and Supplementary Table S-3. The AOP group includes two subgroups characterized by lateral JSN (subgroups 1 and 2) and one subgroup with medial JSN along with pronounced subchondral sclerosis (subgroup 3). Patients with dominant lateral JSN (mean grade of 0.94), lateral subchondral sclerosis (mean grade of 0.98 and 0.89 for lateral femur and lateral tibia, respectively), and frequent high-load exercises (832) exhibited the most rapid progression (subgroup 1). Patients with lateral JSN (mean grade of 1.00) without subchondral sclerosis (mean grade of both 0.03 for lateral femur and lateral tibia) and less high-load exercise (473) engagement also showed fast progression (subgroup 2). Patients with relatively higher-grade medial JSN (mean grade of 0.89) and medial subchondral sclerosis (mean grade of 1.18 and 1.13 for medial femur and medial tibia, respectively) demonstrated accelerated progression (subgroup 3).

Conversely, the SOP group included two subgroups with medial JSN but without medial subchondral sclerosis (subgroups 4 and 5), a subgroup with no JSN but significant osteophyte grade (subgroup 6), and a subgroup with older age and a higher grade of chondrocalcinosis (subgroup 7). The corresponding baseline characteristics and progression proportions to KL grade ≥ 3 in the test set clusters are similarly reproduced (Fig. 4 and Supplementary Table S-4). Radiographic phenotype changes at the 4-year follow-up showed that only those phenotype variables already significantly elevated in each subgroup were aggravated at follow-up (Supplementary Tables S-5 and S-6).

### Long-term survival analysis

**Fig. 6:**
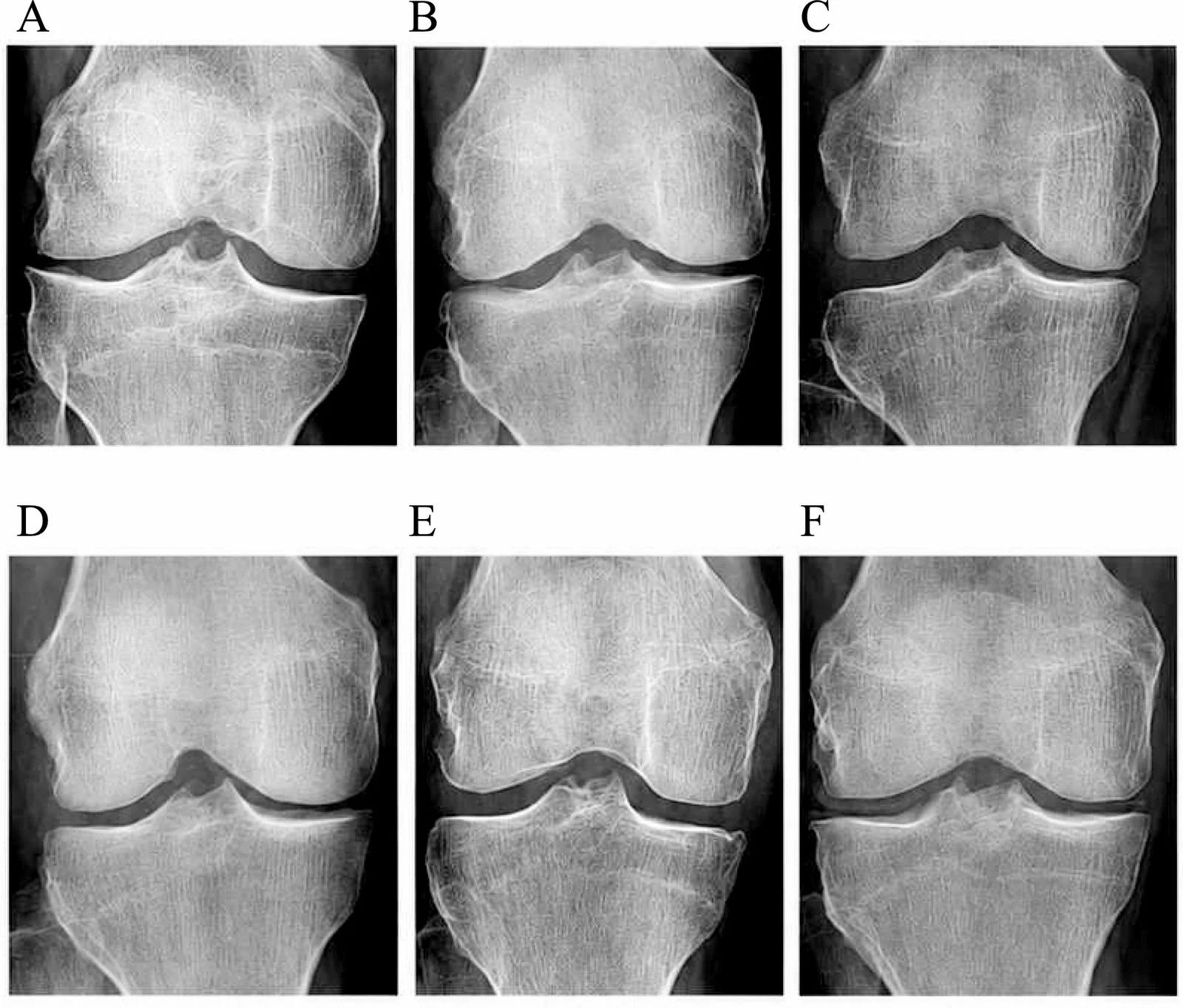
Survival curves of AOP versus SOP group for the **a** training dataset and **b** test dataset. They illustrate differences in cumulative survival over time. Shaded areas represent confidence intervals. *AOP* accelerated osteoarthritis progression group, *SOP* slow osteoarthritis progression group

Overall, time to knee replacement or progression to KL grade 4 did not differ significantly between the training and test sets (log-rank *P* = 0.968; Supplementary Fig. S-1). Within each set, however, the AOP and SOP groups had different survival distributions over 9 years (training set, *P* < 0.001; test set, *P* = 0.049; Fig. 6 and Table 1). Average survival time was 8.3 years (AOP) and 8.5 years (SOP) in the training set, and 8.4 years (AOP) and 8.6 years (SOP) in the test set. Interestingly, in subgroup analysis, subgroup 7, which is included in the SOP group, showed significantly shorter time to knee replacement or progression to KL grade 4 compared with other subgroups in the SOP group: subgroup 7 versus 4 (*P* = 0.011) and 5 (*P* = 0.033) in training set, and subgroup 7 versus 6 (*P* = 0.021) in test set (Supplementary Fig. S-2 and Supplementary Table S-7). This finding highlights the need for tailored approaches even within the SOP group, as certain subgroups may face a more rapid decline than expected.

**Table 1:** Percentage of knee replacement or progression to KL grade 4 (survivals) of AOP group and SOP group for training and test sets

Training set					
	1 year	3 year	5 year	7 year	9 year
AOP group (*n* = 371)	1.000	0.992	0.984	0.940	0.903
SOP group (*n* = 895)	0.999	0.998	0.986	0.970	0.956
Test set					
	1 year	3 year	5 year	7 year	9 year
AOP group (*n* = 153)	1.000	0.993	0.960	0.960	0.914
SOP group (*n* = 377)	1.000	0.989	0.981	0.973	0.955

*AOP* accelerated osteoarthritis progression, *SOP* slow osteoarthritis progression

## Discussion

The most important finding of this study is that patients with early-stage knee OA (KL grade 2) could be grouped into distinct phenotypic subgroups with different progression trajectories. Based on baseline demographic, exercise-related, and radiographic characteristics, the clustering analysis identified subgroups with markedly different rates of progression to KL grades 3 or 4 over time. These results suggest that clinically relevant heterogeneity exists even within the same radiographic grade and that combinations of baseline structural and lifestyle-related factors may help distinguish patients at relatively higher or lower risk of progression. However, because the present study used an unsupervised clustering approach rather than a supervised predictive model, these findings should be interpreted as hypothesis-generating and as a basis for future development of clinically actionable prognostic tools.

Across the seven subgroups, the major phenotypic contrasts involved compartmental JSN with or without subchondral sclerosis, osteophyte-dominant disease without JSN, and an older chondrocalcinosis-dominant pattern. Subgroups 1 and 2 were both lateral JSN-dominant, consistent with the greater structural severity reported in isolated lateral than medial compartment OA [6]. Subgroup 1, however, had lateral subchondral sclerosis, greater high-load exercise exposure, and faster progression than subgroup 2. A similar structural contrast was observed medially: subgroup 3 had medial JSN with sclerosis, whereas subgroup 4 had medial JSN without sclerosis. The contrast suggests that subchondral sclerosis may identify a more progressive lateral JSN phenotype and may be related to abnormal mechanical loading and subchondral remodeling [7]. Subgroup 5, a predominantly male subgroup, combined the medial JSN pattern of subgroup 4 with the osteophyte-dominant pattern of subgroup 6. Despite the greatest exposure to all three exercise-load categories, it showed slow 4-year progression and remained in the SOP group, a pattern that may reflect sex-related differences in OA [18]. Subgroup 6 showed osteophyte-dominant disease without JSN, supporting the distinction between KL grade 2 knees with osteophytes alone and those with JSN, as osteophyte-only knees have shown less cartilage loss [8]. Finally, subgroup 7 was the oldest (mean age, 67.9 years) and chondrocalcinosis-dominant subgroup. Although subgroup 7 was classified as SOP based on 4-year KL progression, it reached KL grade 4 or knee replacement earlier than some other SOP subgroups during longer follow-up. Together with its chondrocalcinosis-dominant features, this supports considering it a distinct phenotype [9, 19, 20].

The distinctive contribution of this study lies in applying a data-driven clustering approach based on radiographic features, exercise-related variables, and demographic characteristics within a clinically focused population of baseline KL grade 2 knees and interpreting the resulting subgroups as clinically recognizable radiographic phenotype patterns. Most machine learning studies focus on progression prediction using biomechanical data [21], biochemical markers [22, 23], genomic data [24, 25], or advanced imaging data [26, 27]. Compared with many of these approaches, our approach used variables that are more readily available in routine clinical datasets, including plain radiographic findings and summarized lifetime exercise exposure. The reduction of 93 exercise-related variables into 3 summary variables was intended to improve the feasibility of incorporating long-term physical activity patterns into the analysis. However, the present unsupervised framework does not establish causal determinants of progression or provide validated individual-level predictive performance. Rather, our findings suggest that combinations of commonly available baseline features may help identify phenotypic subgroups with different progression trajectories and may support future development of more clinically applicable prognostic models.

There are several limitations associated with this study. First, although baseline KL grade 2 status was defined using the OAI central reading dataset, KL grading has only moderate interobserver reliability, particularly at intermediate grades. Therefore, some knees may have been misclassified within the KL grade 2 cohort, which could have introduced additional heterogeneity and influenced the clustering results. However, this limitation also reflects real-world ambiguity in structural OA classification and supports the value of data-driven subgrouping beyond KL grade alone. Second, medial and lateral radiographic features were incorporated into a single clustering framework rather than analyzed separately by compartment. Although this approach was intended to capture overall heterogeneity within KL grade 2 knees, it may have allowed compartment-dominant patterns to influence cluster formation and may have obscured compartment-specific progression pathways. Future studies should evaluate whether separate medial- and lateral-compartment analyses yield more clinically specific phenotypic classifications. Third, several baseline radiographic variables used for clustering, including JSN and osteophyte grades, are components or close correlates of KL grading and radiographic progression; therefore, differences in progression across clusters should be interpreted as associations between baseline radiographic phenotypes and subsequent radiographic progression rather than as independent causal effects of individual radiographic features. Fourth, missing values in several ordinal radiographic variables were handled using median imputation to retain knees with incomplete radiographic data in the analysis. Because median imputation assigns the central observed grade to missing values, it may reduce the influence of rare nonzero radiographic abnormalities on cluster formation. Fifth, the retrospective use of OAI data introduces potential confounding factors that were not fully accounted for, including comorbidities and treatment-related variables that may affect disease progression. In particular, corticosteroid use was not evaluated as an independent factor in the present analysis. BMI was included as a baseline clustering variable, but the unsupervised framework used in this study does not permit interpretation of obesity as an isolated causal contributor to progression. In addition, the exercise variables were derived from retrospectively self-reported lifetime activity data and may, therefore, be affected by recall bias, subjective interpretation, and over- or underestimation of actual exposure. These factors limit causal inference, as the clustering approach identifies associations rather than direct drivers of progression. Sixth, the HDBSCAN parameter settings were intended to favor well-defined clusters, leaving 201 of 1997 knees (10.1%) unassigned and potentially limiting the generalizability of the subgroup findings. Lastly, because there was a significant difference in the average amount of exercise between men and women, it was difficult to isolate the effect of exercise variables alone. The novel categorization of exercises into low, moderate, and high impact groups based on their potential effect on knee OA progression refines the analysis, allowing for a more intuitive evaluation of exercise types. However, if clustering is performed by separating the sexes using a larger dataset, the effect of exercise levels can be examined in more detail.

## Conclusions

The 4-year radiographic progression of knee OA among patients with baseline KL grade 2 varied substantially across phenotypic subgroups defined by demographic data, lifetime exercise patterns, and radiographic features. Unsupervised clustering revealed distinct subgroups with markedly different KL grade progression rates, supporting the classification of early-stage OA into accelerated or slow progression categories. These findings support viewing KL grade 2 knee OA as a heterogeneous radiographic stage comprising clinically interpretable subgroups with different short-term radiographic prognoses.

## Supplementary information

Supplementary Material 1.

## Data Availability

The data used in this study were obtained from the Osteoarthritis Initiative (OAI) database, which is publicly available at https://nda.nih.gov/oai/.

## Abbreviations

OA: Knee osteoarthritis
KL grade: Kellgren and Lawrence grade
OAI: Osteoarthritis Initiative
IRB: Institutional Review Board
OARSI: Osteoarthritis Research Society International
BMI: Body mass index
JSN: Joint space narrowing
UMAP: Uniform manifold approximation and projection
HDBSCAN: Hierarchical density-based spatial clustering of applications with noise
AOP group: Accelerated osteoarthritis progression group
SOP group: Slow osteoarthritis progression group
