# Supplementary material for: Different osteoarthritis progression rates within early-stage knee osteoarthritis: a retrospective cohort study using machine learning cluster analysis from the osteoarthritis initiative database
Source: Knee Surg Relat Res. 2026 Nov 2;38:55. doi: 10.1186/s43019-026-00355-z (PMC13726216; doi:10.1186/s43019-026-00355-z)
Supplement: Supplementary file 1 [file 43019_2026_355_MOESM1_ESM.pdf]

Supplementary Method

As for exercise-related variables, we employed a comprehensive approach to quantify the lifetime exercise load among participants by examining their engagement in specific exercises.

In the OAI cohort, the exercise variables are extensively categorized into 93 distinct variables, making the utilization of these exercise variables in research challenging. Consequently, few studies in the existing literature on machine learning models for predicting osteoarthritis have incorporated these exercise variables due to the complexity involved. When exercise variables were used, they were typically simplified without detailed consideration of the specific types or frequencies of exercises. Unlike these previous approaches, our approach aims to capture the impact of various exercises on OA risk more accurately. Instead of using oversimplified frequency variables alone, we attempted to calculate the lifetime cumulative number of exercise sessions.

To quantify the cumulative impact of exercises over an individual's lifespan, we calculated the Lifelong Engagement Times (LET) and incorporated the Competitiveness Factor (CF) for each age group and activity level.

Specifically:

- Lifelong Engagement Times (LET) is calculated using a straightforward formula: (Number of Years) x (Number of Months per Year) x (Number of Times per Month) engaged in activities.
- Competitiveness Factor (CF) enhances the Lifelong Engagement Times, applying a multiplier of 1.5 for competitive exercises (those answering 'yes' to performing competitively) and 1 for non-competitive ones. This adjustment recognizes the higher intensity and impact of competitive activities on the body.

The variables were surveyed for each exercise that patients frequently performed among 37 different exercises. With reference to the loading concepts described by Buckwalter[1] and Schirò et al.,[2] the study team developed an operational classification of the 37 exercises into low-, moderate-, and high-load categories for the present study.

- Low load exercise:

  Elliptical trainer, Fishing, Gardening or yardwork, Golfing with a cart, Golfing without a cart, Handball, Snow skiing (downhill), Stairmaster or walking up and down stairs, Swimming, Tai Chi, Water aerobics, Yoga, **Carpentry, *Gymnastics, *Jumping rope.*

- Moderate load exercise:

  Bicycling or spinning, Bowling, Canoeing, rowing or kayaking, Jogging or running (outdoor or indoor treadmill or track), Roller blading or skating, Sailing, Snow skiing (cross-country or Nordic track), Strength or weight training, Table tennis or Ping Pong, **Aerobic dance or step aerobics, *Dancing, *Ice skating.*

- High load exercise: Badminton, Baseball or softball, Basketball, Football, Racquetball or squash, Soccer, Tennis (doubles), Tennis (singles), Volleyball, **Hockey (ice or field)*

Items marked with an asterisk were not explicitly categorized in the cited literature and were assigned by the study team to the operational load categories by analogy according to their loading characteristics.

Exercise Categorization Table

Exercises not explicitly categorized in the cited literature	Category	Rationale
Aerobic dance or step aerobics	Moderate	low-impact aerobics is specified in low-loading, inferring high-impact aerobics(aerobic dance etc.) has more loads.
Carpentry	Low	do not use knee as much as other activities
Dancing	Moderate	similar to aerobic dance or step aerobics
Gymnastics	Low	do not use knee as much as other activities
Hockey (ice or field)	High	similar to soccer
Ice skating	Moderate	similar to in-line skating
Jumping rope	Low	similar to walking

We encountered the challenge of accurately quantifying exercise engagement due to the categorical nature of key variables. These variables were surveyed as categorical options (e.g., 'Number of Years': 1 for '1-2 years', 2 for '3-4 years', 3 for '5-7 years'), rather than continuous data points (e.g. N years), necessitating a 'midpoint method' for our calculations. This method calculates the representative value for each category (e.g., 1.5 years for '1-2 years') to estimate exercise duration and frequency more precisely.

For instance, consider a participant who has engaged in soccer and basketball, both high load exercises, and swimming, a low load exercise.

- Basketball (Exercise 1): Classified as a high load exercise, the individual engaged in basketball competitively, applying a Competitiveness Factor 1 (CF1) of 1.5. Utilizing midpoint values, their engagement spanned an average of 8 years (categorized as 6-10 years, year_1 (Y1) = 8), with activity for about 6.5 months each year (5-8 months, month_1 (M1) = 6.5), and participation 6 times per month (4-8 times, time_1 (T1) = 6). Therefore, LET1 * CF1= (Y1 * M1 * T1) * CF1 = 468.
- Soccer (Exercise 2): Also a high load exercise but participated in non-competitively, thus assigned a CF2 of 1. If the individual engaged for the same duration as basketball (Y1 = Y2, M1 = M2, T1 = T2), their soccer involvement would similarly be

quantified using the respective midpoints, but without the enhanced CF due to the lack of competitive play. Therefore, LET2 * CF2= (Y2 * M2 * T2) * CF2 = 312.

- Swimming (Exercise 3): Categorized under low load exercises, swimming, assumed to be engaged in non-competitively, is assigned a CF3 of 1. Assuming engagement for 3 years (1-5 years, Y3 = 3), with 2.5 months of activity each year (1-4 months, M3 = 2.5), and participating 2 times per month (1-3 times, T3 = 2), swimming's impact is quantified with these lower intensity and frequency parameters. Therefore, LET3 * CF3 = (Y3 * M3 * T3) * CF3 = 15.

For high load exercises (soccer and basketball), we calculate the cumulative impact as: 468 + 312 = 780. For the low load exercise (swimming), the calculation is: 15. This approach allows us to account for both the duration and intensity of engagement, offering a nuanced view of exercise's cumulative impact on health.

1. Buckwalter JA. Sports, joint injury, and posttraumatic osteoarthritis. J Orthop Sports Phys Ther 2003;33(10):578-88. (In eng). DOI: 10.2519/jospt.2003.33.10.578.
2. Schirò S, Foreman SC, Joseph GB, et al. Impact of different physical activity types on knee joint structural degeneration assessed with 3-T MRI in overweight and obese subjects: data from the osteoarthritis initiative. Skeletal Radiol 2021;50(7):1427-1440. (In eng). DOI: 10.1007/s00256-020-03642-2.

Supplementary Figures

Supplementary Figure S1. Survival curve until knee replacement or progression to KL grade 4 of train and test datasets. Shading represents 95% confidence interval. Log-rank test showed there is no statistically significant difference in survival distributions between the train and test datasets (p-value: 0.968).

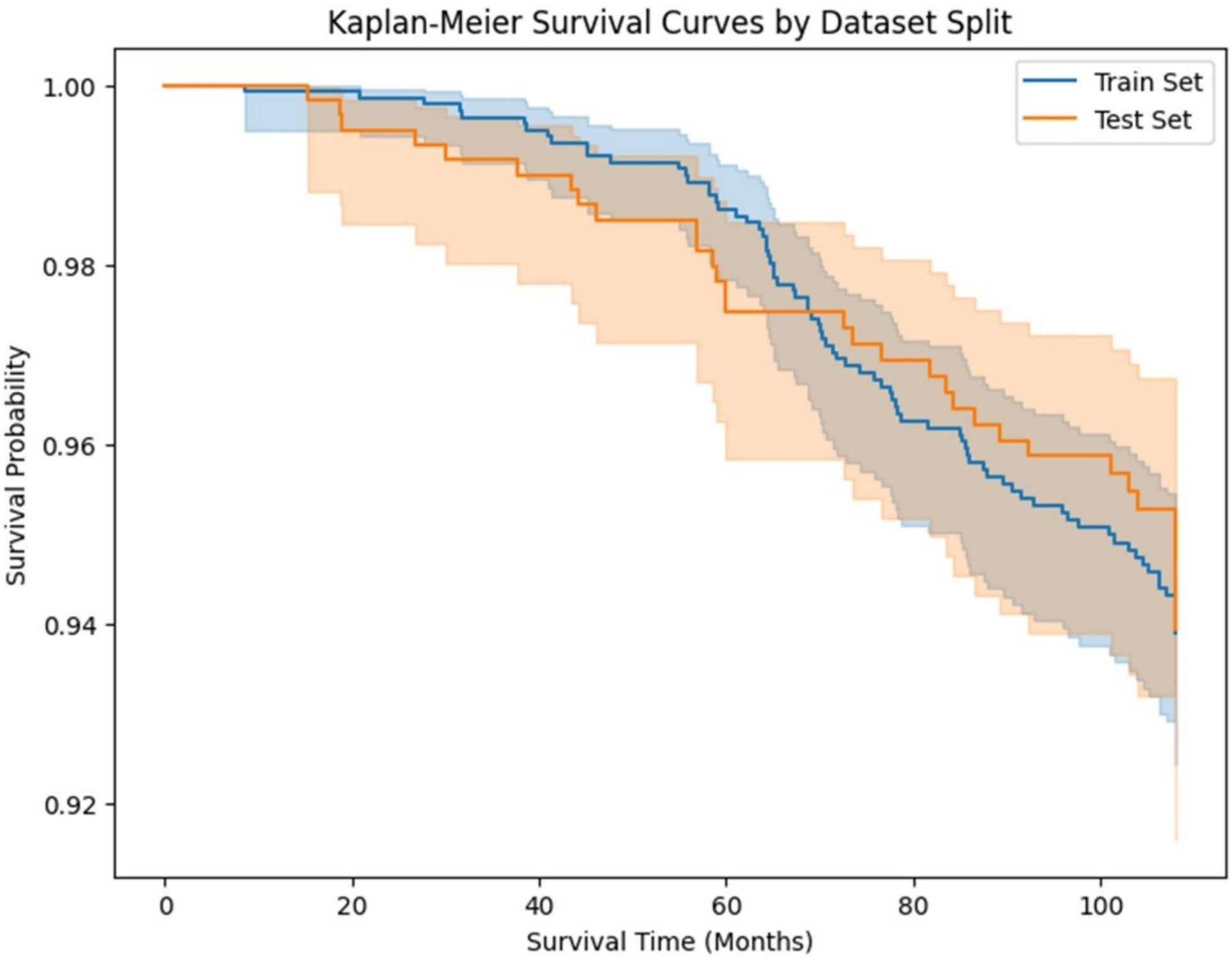

Supplementary Figure S2. Survival curves of each subgroup until knee replacement or progression to KL grade 4 for the (A) train dataset and (B) test dataset. Pairwise log-rank tests showed significant survival distinctions between AOP group and SOP group: subgroup 1 vs. 4 (P<0.001), 5 (P<0.001), and 6 (P=0.005); subgroup 2 vs. 4 (P=0.024); subgroup 3 vs. 4 (P=0.012) and 5 (P=0.026) in training set. In test set, similar significant differences were shown between AOP group and SOP group: subgroup 1 vs. 4 (P=0.004), 5 (P=0.012), and 6 (P<0.001). Interestingly, subgroup 7, which is included in SOP group, showed significantly faster knee replacement compared to other subgroups in SOP group: subgroup 7 vs. 4 (P=0.011) and 5 (P=0.033) in training set, and subgroup 7 vs. 6 (P=0.021) in test set.

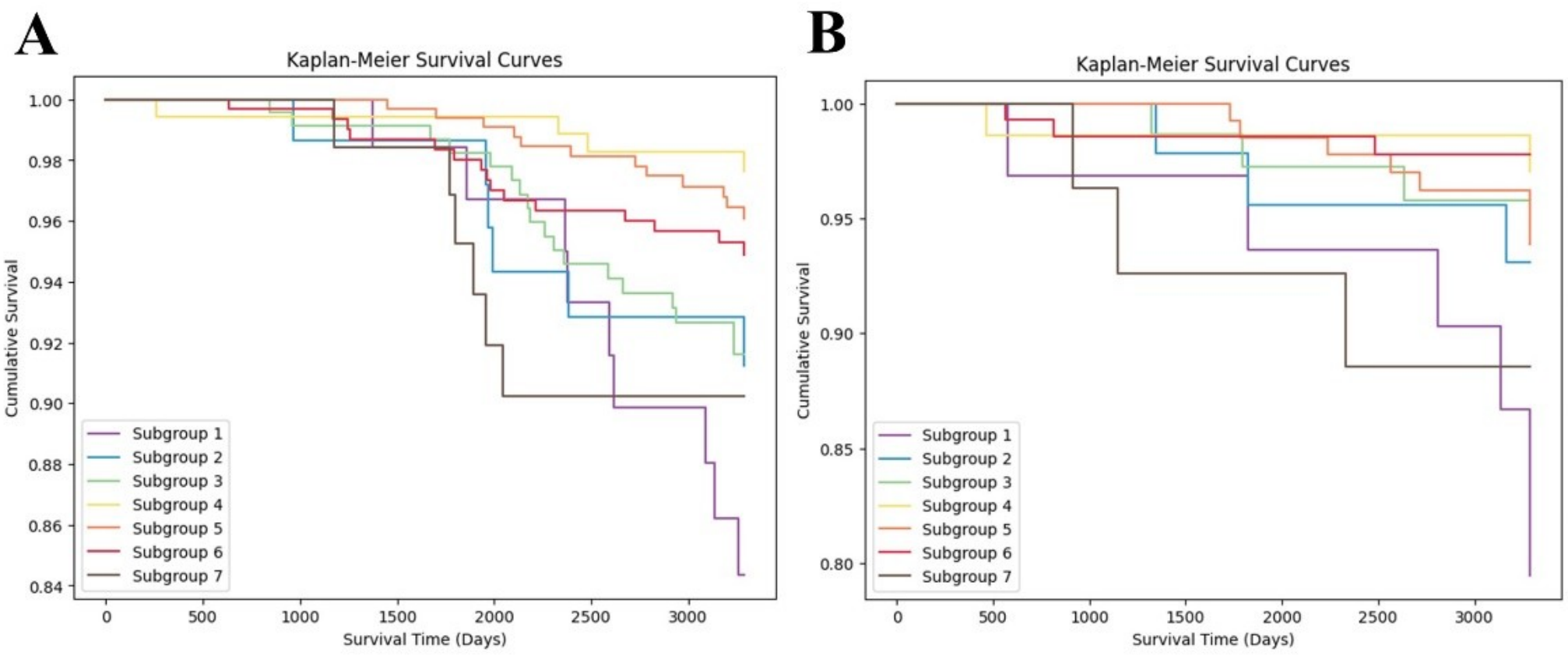

Supplementary Figure S3. Outcome-independent sensitivity analysis of UMAP-HDBSCAN hyperparameters. (A) DBCV and non-noise silhouette coefficients for the 482 training-set refits that produced seven clusters. Dark blue points indicate high concordance with the primary clustering solution (ARI ≥ 0.90), and the red star indicates the primary solution. (B) Number of high-concordance seven-cluster refits at each value of UMAP n_neighbors and min_dist and HDBSCAN min_cluster_size and training-set min_samples. For each displayed parameter value, the bar is a marginal count pooled over all combinations of the other three hyperparameters; it does not represent a one-at-a-time perturbation around the primary parameter tuple. Red outlines indicate the parameter values used in the primary analysis. DBCV was calculated in the fitted two-dimensional UMAP space; the silhouette coefficient excluded observations labeled as noise; and ARI included noise as a label. ARI was used only as a post hoc measure of concordance and not as an intrinsic validity metric or parameter-selection criterion. No progression or survival outcome was used in the sensitivity analysis.

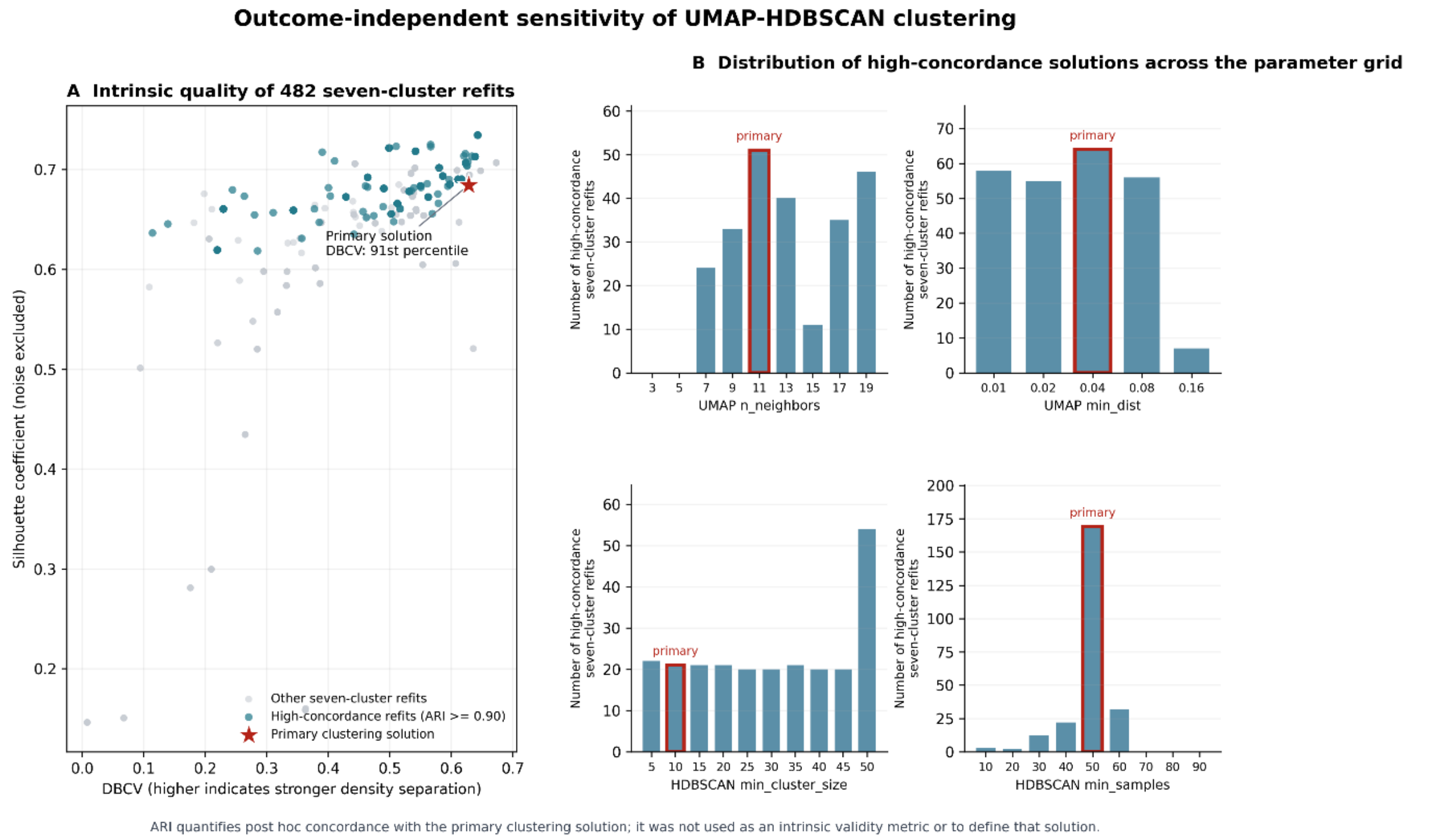

Supplementary Figure S4. Pairwise cosine similarity matrix between training- and test-set cluster centroids. Cluster labels generated independently by HDBSCAN have no inherent correspondence between datasets. Therefore, matching was performed using the independent raw HDBSCAN labels before relabeling. The 24 baseline clustering variables were standardized using the training-set means and standard deviations, and the same transformation was applied to the test set. All 49 pairwise cosine similarities between training- and test-set cluster centroids were calculated, and the one-to-one assignment maximizing total similarity was determined using the Hungarian algorithm. Rows are displayed after relabeling the test-set clusters according to this outcome-independent assignment. Black boxes indicate the matched pairs. No progression or survival outcome was used in the matching procedure.

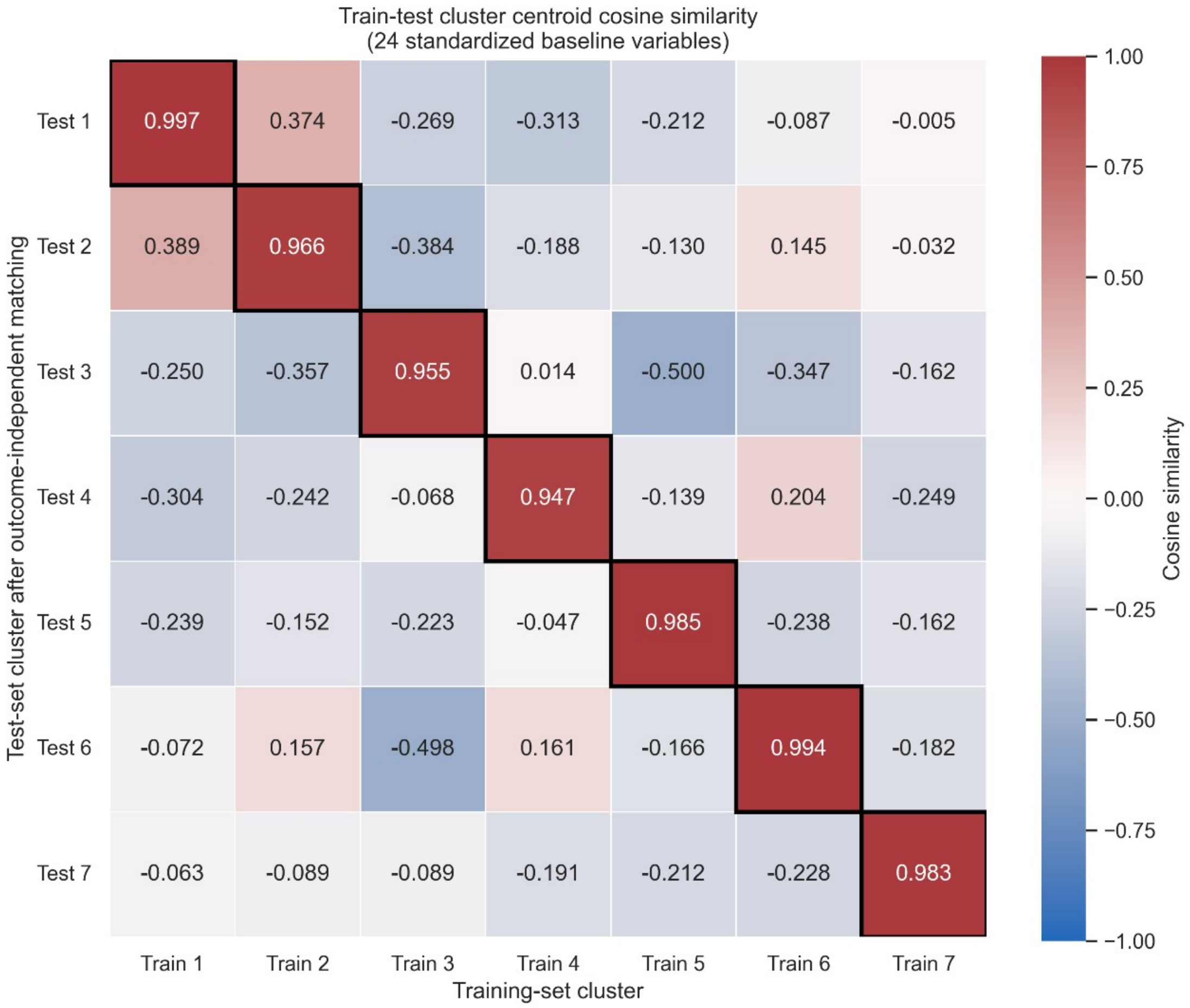

Supplementary Table S1. Patient Characteristics of Training and Test set*

Variable	All Patients (N = 1997)	Training set (N = 1397)	Test set (N = 600)	P	Missing
Follow-up, mo	103.3±13.7	103.1±13.9	103.6±13.1	0.497	0 (0)
Male sex	742 (37)	534 (38)	208 (35)	0.147	0 (0)
Age at baseline X-ray	61.4±8.7	61.5±8.6	61.2±9.0	0.44	0 (0)
Side of knee (Right)	1032 (52)	727 (52)	305 (51)	1	0 (0)
Body mass index				0.908	0 (0)
18.5 - 24.9	358 (17.9%)	249 (17.8%)	109 (18.2%)		
25 - 29.9	732 (36.7%)	516 (36.9%)	216 (36.0%)		
> 30	907 (45.4%)	632 (45.2%)	275 (45.8%)		
Low load exercise	1809.7±3046.7	1864.2±3131.2	1683.0±2838.9	0.814	0 (0)
Moderate load exercise	1882.2±3532.2	1924.1±3638.5	1784.5±3271.9	0.879	0 (0)
High load exercise	654.9±1936.4	662.1±1943.3	638.1±1921.7	0.503	0 (0)
Joint space narrowing					
Medial				0.742	0 (0)
0	957 (47.9%)	673 (48.2%)	284 (47.3%)		
1	1039 (52.0%)	723 (51.8%)	316 (52.7%)		
2	1 (0.1%)	1 (0.1%)	0 (0.0%)		
Lateral†	242 (12)	155 (11)	87 (14)	0.037	0 (0)
Osteophyte					
Femur, Medial				0.231	0 (0)
0	980 (49.1%)	695 (49.7%)	285 (47.5%)		
1	664 (33.2%)	467 (33.4%)	197 (32.8%)		
2	210 (10.5%)	135 (9.7%)	75 (12.5%)		
3	143 (7.2%)	100 (7.2%)	43 (7.2%)		
Tibia, Medial				0.965	0 (0)
0	554 (27.7%)	382 (27.3%)	172 (28.7%)		
1	1260 (63.1%)	895 (64.1%)	365 (60.8%)		
2	150 (7.5%)	95 (6.8%)	55 (9.2%)		
3	33 (1.7%)	25 (1.8%)	8 (1.3%)		
Femur, Lateral				0.182	0 (0)
0	1059 (53.0%)	756 (54.1%)	303 (50.5%)		
1	679 (34.0%)	463 (33.1%)	216 (36.0%)		
2	177 (8.9%)	118 (8.4%)	59 (9.8%)		
3	82 (4.1%)	60 (4.3%)	22 (3.7%)		
Tibia, Lateral				0.371	0 (0)
0	1112 (55.7%)	785 (56.2%)	327 (54.5%)		
1	705 (35.3%)	493 (35.3%)	212 (35.3%)		
2	104 (5.2%)	67 (4.8%)	37 (6.2%)		
3	76 (3.8%)	52 (3.7%)	24 (4.0%)		
Subchondral sclerosis					
Femur, Medial				0.935	178 (9)
0	1369 (68.6%)	961 (68.8%)	408 (68.0%)		
1	401 (20.1%)	283 (20.3%)	118 (19.7%)		
2	48 (2.4%)	34 (2.4%)	14 (2.3%)		
3	1 (0.1%)	0 (0.0%)	1 (0.2%)		
Tibia, Medial				0.906	178 (9)
0	1326 (66.4%)	930 (66.6%)	396 (66.0%)		
1	439 (22.0%)	312 (22.3%)	127 (21.2%)		
2	53 (2.7%)	36 (2.6%)	17 (2.8%)		
3	1 (0.1%)	0 (0.0%)	1 (0.2%)		
Femur, Lateral				0.407	178 (9)
0	1720 (86.1%)	1212 (86.8%)	508 (84.7%)		
1	90 (4.5%)	62 (4.4%)	28 (4.7%)		
2	8 (0.4%)	4 (0.3%)	4 (0.7%)		
3	1 (0.1%)	0 (0.0%)	1 (0.2%)		
Tibia, Lateral				0.421	178 (9)
0	1739 (87.1%)	1225 (87.7%)	514 (85.7%)		
1	69 (3.5%)	46 (3.3%)	23 (3.8%)		
2	11 (0.6%)	7 (0.5%)	4 (0.7%)		
Chondrocalcinosis					
Medial†	103 (5)	73 (5)	30 (5)	0.95	1 (0)
Lateral†	103 (5)	72 (5)	31 (5)	1	1 (0)

* Data are reported as mean ± SD or N(%). Text in bold indicate significant differentiating factors among subgroups.

† Since the OA phenotype has only grade 0 and 1, only data of grade 1 is presented.

Supplementary Table S2. Characteristics of Patients who Did or Did Not Progress to KL-grade 3 or 4 in 48 Months of Follow Up*

Variable	All Patients (N = 1997)	Progressed (N = 294)	Did Not Progress (N = 1703)	P-value	Missing
Follow-up, mo	103.3±13.7	102.1±15.4	103.5±13.3	0.237	0 (0)
Male sex	742 (37)	99 (34)	643 (38)	0.208	0 (0)
Age at baseline X-ray	61.4±8.7	61.8±8.2	61.4±8.8	0.344	0 (0)
Side of knee (Right)	1032 (52)	164 (56)	868 (51)	1	0 (0)
Body mass index				<0.001	0 (0)
18.5 - 24.9	358 (17.9%)	37 (12.6%)	321 (18.8%)		
25 - 29.9	732 (36.7%)	93 (31.6%)	639 (37.5%)		
> 30	907 (45.4%)	164 (55.8%)	743 (43.6%)		
Low load exercise	1809 ± 3046	1823 ± 3228	1807 ± 3015	0.254	0 (0)
Moderate load exercise	1882 ± 3532	1499 ± 2882	1948 ± 3629	0.1	0 (0)
High load exercise	654 ± 1936	629 ± 2251	659 ± 1877	0.034	0 (0)
Joint space narrowing					
Medial				0.01	0 (0)
0	957 (47.9%)	121 (41.2%)	836 (49.1%)		
1	1039 (52.0%)	172 (58.5%)	867 (50.9%)		
2	1 (0.1%)	1 (0.3%)	0 (0.0%)		
Lateral†	242 (12)	79 (27)	163 (10)	<0.001	0 (0)
Osteophyte					
Femur, Medial				0.017	0 (0)
0	980 (49.1%)	128 (43.5%)	852 (50.0%)		
1	664 (33.2%)	103 (35.0%)	561 (32.9%)		
2	210 (10.5%)	32 (10.9%)	178 (10.5%)		
3	143 (7.2%)	31 (10.5%)	112 (6.6%)		
Tibia, Medial				0.771	0 (0)
0	554 (27.7%)	85 (28.9%)	469 (27.5%)		
1	1260 (63.1%)	181 (61.6%)	1079 (63.4%)		
2	150 (7.5%)	22 (7.5%)	128 (7.5%)		
3	33 (1.7%)	6 (2.0%)	27 (1.6%)		
Femur, Lateral				0.006	0 (0)
0	1059 (53.0%)	139 (47.3%)	920 (54.0%)		
1	679 (34.0%)	100 (34.0%)	579 (34.0%)		
2	177 (8.9%)	36 (12.2%)	141 (8.3%)		
3	82 (4.1%)	19 (6.5%)	63 (3.7%)		
Tibia, Lateral				<0.001	0 (0)
0	1112 (55.7%)	137 (46.6%)	975 (57.3%)		
1	705 (35.3%)	121 (41.2%)	584 (34.3%)		
2	104 (5.2%)	27 (9.2%)	77 (4.5%)		
3	76 (3.8%)	9 (3.1%)	67 (3.9%)		
Subchondral sclerosis					
Femur, Medial				<0.001	178 (9)
0	1369 (68.6%)	165 (56.1%)	1204 (70.7%)		
1	401 (20.1%)	91 (31.0%)	310 (18.2%)		
2	48 (2.4%)	15 (5.1%)	33 (1.9%)		
3	1 (0.1%)	0 (0.0%)	1 (0.1%)		
Tibia, Medial				<0.001	178 (9)
0	1326 (66.4%)	168 (57.1%)	1158 (68.0%)		
1	439 (22.0%)	86 (29.3%)	353 (20.7%)		
2	53 (2.7%)	17 (5.8%)	36 (2.1%)		
3	1 (0.1%)	0 (0.0%)	1 (0.1%)		
Femur, Lateral				<0.001	178 (9)
0	1720 (86.1%)	224 (76.2%)	1496 (87.8%)		
1	90 (4.5%)	41 (13.9%)	49 (2.9%)		
2	8 (0.4%)	6 (2.0%)	2 (0.1%)		
3	1 (0.1%)	0 (0.0%)	1 (0.1%)		
Tibia, Lateral					178 (9)
0	1739 (87.1%)	229 (77.9%)	1510 (88.7%)	<0.001	
1	69 (3.5%)	36 (12.2%)	33 (1.9%)	<0.001	
2	11 (0.6%)	6 (2.0%)	5 (0.3%)	<0.001	
Chondrocalcinosis					
Medial†	103 (5)	10 (3)	93 (5)	0.216	1 (0)
Lateral†	103 (5)	13 (4)	90 (5)	0.7	1 (0)

* Data are reported as mean ± SD or N(%). Text in bold indicate significant differentiating factors among subgroups.
† Since the OA phenotype has only grade 0 and 1, only data of grade 1 is presented.

Supplementary Table S3. Characteristics of the Subgroups in the Training Set*

	All Training Set	AOP group†			SOP group†					
		**Subgroup 1**	**Subgroup 2**	**Subgroup 3**	**Subgroup 4**	**Subgroup 5**	**Subgroup 6**	**Subgroup 7**		
	(N = 1266)	(N = 63)	(N = 75)	(N = 233)	(N = 180)	(N = 343)	(N = 308)	(N = 64)	P-value	Missing
Follow-up, mo	103.1 (14.0)	102.3 (16.5)	101.1 (16.9)	102.7 (14.4)	104.1 (12.0)	102.1 (15.7)	104.5 (11.5)	103.5 (12.9)	0.266	0 (0)
Progression to KL 3 or 4‡	184 (15)	**33 (52)**	**19 (25)**	**56 (24)**	20 (11)	30 (9)	19 (6)	7 (11)	<0.001	0 (0)
Male sex	509 (40)	21 (33)	29 (39)	87 (37)	**1 (1)**	**338 (99)**	**2 (1)**	**31 (48)**	<0.001	0 (0)
Age at baseline X-ray	61.3 (8.6)	63.6 (9.0)	61.0 (9.4)	61.0 (8.4)	62.5 (8.4)	60.7 (8.9)	59.9 (8.0)	**67.9 (7.3)**	<0.001	0 (0)
Side of knee (Right)	655 (52)	36 (57)	36 (48)	114 (49)	108 (60)	175 (51)	150 (49)	36 (56)	0.94	0 (0)
Body mass index									0.002	0 (0)
18.5 - 24.9	228 (18)	**15 (24)**	9 (12)	39 (17)	39 (22)	42 (12)	61 (20)	**23 (36)**		
25 - 29.9	472 (37)	25 (40)	31 (41)	72 (31)	57 (32)	154 (45)	109 (35)	24 (38)		
> 30	566 (45)	23 (37)	35 (47)	**122 (52)**	84 (47)	147 (43)	138 (45)	17 (27)		
Low load exercise	1942 (3205)	1261 (2657)	1892 (3285)	2135 (3499)	1838 (3291)	2272 (3259)	1581 (2742)	2234 (3810)	0.002	0 (0)
Moderate load exercise	2023 (3761)	1337 (2576)	1526 (2961)	1560 (2580)	1572 (2426)	**3180 (5428)**	1594 (2836)	2103 (4057)	0.001	0 (0)
High load exercise	702 (2014)	**832 (2683)**	**473 (1057)**	**807 (1880)**	**183 (568)**	**1431 (3028)**	**175 (477)**	552 (1753)	<0.001	0 (0)
Joint Space Narrowing										
Medial§	614 (48)	4 (6)	6 (8)	**207 (89)**	**180 (100)**	185 (54)	**0 (0)**	32 (50)	<0.001	0 (0)
Lateral§	150 (12)	**59 (94)**	**75 (100)**	4 (2)	0 (0)	0 (0)	**0 (0)**	12 (19)	<0.001	0 (0)
Osteophyte										
Femur, Medial									<0.001	0 (0)
0	639 (50)	**38 (60)**	**46 (61)**	64 (27)	67 (37)	**261 (76)**	129 (42)	34 (53)		
1	425 (34)	14 (22)	15 (20)	**98 (42)**	**84 (47)**	67 (20)	**126 (41)**	21 (33)		
2	122 (10)	5 (8)	6 (8)	**35 (15)**	**24 (13)**	8 (2)	**42 (14)**	2 (3)		
3	80 (6)	6 (10)	8 (11)	**36 (15)**	5 (3)	7 (2)	11 (4)	7 (11)		
Tibia, Medial									<0.001	0 (0)
0	357 (28)	**31 (49)**	**33 (44)**	21 (9)	37 (21)	99 (29)	109 (35)	**27 (42)**		
1	800 (63)	29 (46)	36 (48)	**173 (74)**	**132 (73)**	228 (66)	168 (55)	34 (53)		
2	88 (7)	1 (2)	4 (5)	**27 (12)**	11 (6)	16 (5)	**27 (9)**	2 (3)		
3	21 (2)	2 (3)	2 (3)	**12 (5)**	0 (0)	0 (0)	4 (1)	1 (2)		
Femur, Lateral									<0.001	0 (0)
0	688 (54)	20 (32)	27 (36)	**147 (63)**	**123 (68)**	190 (55)	149 (48)	32 (50)		
1	428 (34)	22 (35)	**33 (44)**	53 (23)	49 (27)	134 (39)	**116 (38)**	21 (33)		
2	104 (8)	**11 (17)**	**8 (11)**	21 (9)	6 (3)	17 (5)	**36 (12)**	5 (8)		
3	46 (4)	**10 (16)**	**7 (9)**	12 (5)	2 (1)	2 (1)	7 (2)	6 (9)		
Tibia, Lateral									<0.001	0 (0)
0	711 (56)	16 (25)	29 (39)	**145 (62)**	**124 (69)**	**232 (68)**	140 (45)	25 (39)		
1	457 (36)	**31 (49)**	**29 (39)**	69 (30)	44 (24)	102 (30)	**146 (47)**	**36 (56)**		
2	60 (5)	**11 (17)**	**9 (12)**	7 (3)	9 (5)	7 (2)	15 (5)	2 (3)		
3	38 (3)	**5 (8)**	**8 (11)**	12 (5)	3 (2)	2 (1)	7 (2)	1 (2)		
Subchondral sclerosis										
Femur, Medial									<0.001	111 (9)
0	895 (71)	59 (94)	63 (84)	2 (1)	153 (85)	305 (89)	270 (88)	43 (67)		
1	230 (18)	4 (6)	2 (3)	**204 (88)**	6 (3)	1 (0)	3 (1)	10 (16)		
2	30 (2)	0 (0)	0 (0)	**27 (12)**	0 (0)	1 (0)	0 (0)	2 (3)		
Tibia, Medial									<0.001	111 (9)
0	880 (70)	57 (90)	63 (84)	12 (5)	157 (87)	277 (81)	270 (88)	44 (69)		
1	245 (19)	6 (10)	2 (3)	**193 (83)**	2 (1)	29 (8)	3 (1)	10 (16)		
2	30 (2)	0 (0)	0 (0)	**28 (12)**	0 (0)	1 (0)	0 (0)	1 (2)		
Femur, Lateral									<0.001	111 (9)
0	1090 (86)	5 (8)	65 (87)	230 (99)	159 (88)	307 (90)	273 (89)	51 (80)		
1	61 (5)	**54 (86)**	0 (0)	3 (1)	0 (0)	0 (0)	0 (0)	4 (6)		
2	4 (0)	**4 (6)**	0 (0)	0 (0)	0 (0)	0 (0)	0 (0)	0 (0)		
Tibia, Lateral									<0.001	111 (9)
0	1104 (87)	14 (22)	65 (87)	232 (100)	159 (88)	307 (90)	273 (89)	54 (84)		

1	44 (3)	**42 (67)**	0 (0)	1 (0)	0 (0)	0 (0)	0 (0)	1 (2)		
2	7 (1)	**7 (11)**	0 (0)	0 (0)	0 (0)	0 (0)	0 (0)	0 (0)		
Chondrocalcinosis										
Medial§	66 (5)	0 (0)	0 (0)	1 (0)	0 (0)	1 (0)	0 (0)	**64 (100)**	<0.001	1 (0)
Lateral§	68 (5)	0 (0)	0 (0)	2 (1)	0 (0)	2 (1)	0 (0)	**64 (100)**	<0.001	1 (0)

* Data are reported as mean (SD) or N(%). Text in bold indicate significant differentiating factors among subgroups.

† AOP group, Accelerated Osteoarthritis Progression group; SOP group, Slow Osteoarthritis Progression group

‡ KL, Kellgren and Lawrence grade

§ Since the OA phenotype has only grade 0 and 1, only data of grade 1 is presented.

Subgroups 1, 2, and 3 showed higher progression rates (52%, 25% and 24%, respectively) than the average (15%), while subgroups 4, 5, 6, and 7 showed lower progression rates (11%, 9%, 6% and 11%, respectively). Subgroup 1 showed deterioration in lateral JSN (94% in lateral JSN grade 1), osteophytes (16% in lateral femur osteophyte grade 3) and subchondral sclerosis (86% in lateral femur subchondral sclerosis grade 1), while subgroup 2, initially lacked lateral subchondral sclerosis (0% in lateral femur subchondral sclerosis grade 1). Subgroup 3 showed deterioration in medial JSN (89% in lateral JSN grade 1), osteophytes (15% in medial femur osteophyte grade 3) and subchondral sclerosis (88% in medial femur subchondral sclerosis grade 1), while subgroup 4, initially lacked lateral subchondral sclerosis (3% in medial femur subchondral sclerosis grade 1). Subgroup 5 is characterized by male participants with a medial-dominant OA pattern. Subgroup 6 showed no JSN but a predominance of osteophytes regardless of location (medial/lateral, femur/tibia). Subgroup 7, the oldest group (67.9 years), has chondrocalcinosis (100% in both medial and lateral chondrocalcinosis).

Supplementary Table S4. Characteristics of the Subgroups in the Test Set*

	All Test Set	AOP group†			SOP group†					
		Subgroup 1	Subgroup 2	Subgroup 3	Subgroup 4	Subgroup 5	Subgroup 6	Subgroup 7		
	(N = 530)	(N = 32)	(N = 46)	(N = 75)	(N = 73)	(N = 138)	(N = 139)	(N = 27)	P-value	Missing
Follow-up, mo	103.9 (12.4)	102.8 (13.9)	103.3 (13.5)	100.8 (16.9)	105.4 (8.8)	105.2 (10.4)	104.2 (12.3)	102.7 (13.0)	0.243	0 (0)
Progression to KL 3 or 4‡	67 (13)	**15 (47)**	**10 (22)**	11 (15)	11 (15)	14 (10)	4 (3)	2 (7)	<0.001	0 (0)
Male sex	178 (34)	11 (34)	14 (30)	**4 (5)**	**1 (1)**	**136 (99)**	**1 (1)**	11 (41)	<0.001	0 (0)
Age at baseline X-ray	61.2 (8.9)	62.7 (9.1)	64.1 (10.7)	62.2 (9.1)	59.8 (8.7)	60.8 (8.7)	59.4 (8.3)	**67.8 (4.9)**	<0.001	0 (0)
Side of knee (Right)	273 (52)	17 (53)	20 (43)	34 (45)	46 (63)	70 (51)	71 (51)	15 (56)	0.967	0 (0)
Body mass index									0.14	0 (0)
18.5 - 24.9	99 (19)	6 (19)	**13 (28)**	12 (16)	13 (18)	17 (12)	31 (22)	**7 (26)**		
25 - 29.9	191 (36)	15 (47)	17 (37)	22 (29)	18 (25)	70 (51)	41 (29)	8 (30)		
> 30	240 (45)	11 (34)	16 (35)	**41 (55)**	**42 (58)**	51 (37)	67 (48)	12 (44)		
Low load exercise	1709 (2791)	1797 (3438)	2182 (3442)	1130 (2149)	1365 (2329)	**2307 (3037)**	1560 (2708)	1043 (2020)	<0.001	0 (0)
Moderate load exercise	1794 (3297)	1823 (3833)	1544 (2204)	807 (1585)	1545 (2631)	**3054 (4818)**	1443 (2407)	967 (1737)	0.001	0 (0)
High load exercise	627 (1949)	**859 (2019)**	**570 (1487)**	**166 (815)**	**229 (750)**	**1661 (3262)**	**138 (354)**	**32 (106)**	<0.001	0 (0)
Joint Space Narrowing										
Medial§	251 (47)	1 (3)	6 (13)	**67 (89)**	**73 (100)**	88 (64)	**0 (0)**	16 (59)	<0.001	0 (0)
Lateral§	82 (15)	**32 (100)**	**46 (100)**	0 (0)	0 (0)	0 (0)	**0 (0)**	4 (15)	<0.001	0 (0)
Osteophyte										
Femur, Medial									<0.001	0 (0)
0	261 (49)	24 (75)	26 (57)	18 (24)	27 (37)	96 (70)	54 (39)	16 (59)		
1	169 (32)	4 (12)	10 (22)	**32 (43)**	**28 (38)**	32 (23)	**56 (40)**	7 (26)		
2	64 (12)	1 (3)	6 (13)	**13 (17)**	9 (12)	9 (7)	**23 (17)**	3 (11)		
3	36 (7)	3 (9)	4 (9)	**12 (16)**	**9 (12)**	1 (1)	6 (4)	1 (4)		
Tibia, Medial									<0.001	0 (0)
0	161 (30)	17 (53)	22 (48)	9 (12)	19 (26)	36 (26)	54 (39)	4 (15)		
1	317 (60)	13 (41)	22 (48)	46 (61)	**49 (67)**	**93 (67)**	72 (52)	**22 (81)**		
2	47 (9)	2 (6)	2 (4)	**18 (24)**	4 (5)	9 (7)	11 (8)	1 (4)		
3	5 (1)	0 (0)	0 (0)	**2 (3)**	1 (1)	0 (0)	2 (1)	0 (0)		
Femur, Lateral									<0.001	0 (0)
0	267 (50)	6 (19)	23 (50)	43 (57)	40 (55)	76 (55)	63 (45)	16 (59)		
1	195 (37)	**14 (44)**	13 (28)	20 (27)	27 (37)	54 (39)	**60 (43)**	7 (26)		
2	54 (10)	**11 (34)**	**8 (17)**	8 (11)	6 (8)	7 (5)	11 (8)	3 (11)		
3	14 (3)	1 (3)	2 (4)	4 (5)	0 (0)	1 (1)	5 (4)	1 (4)		
Tibia, Lateral									<0.001	0 (0)
0	283 (53)	6 (19)	16 (35)	51 (68)	42 (58)	92 (67)	63 (45)	13 (48)		
1	194 (37)	**17 (53)**	**25 (54)**	19 (25)	27 (37)	40 (29)	**58 (42)**	8 (30)		
2	34 (6)	**6 (19)**	3 (7)	5 (7)	3 (4)	2 (1)	11 (8)	4 (15)		
3	19 (4)	**3 (9)**	2 (4)	0 (0)	1 (1)	4 (3)	7 (5)	2 (7)		
Subchondral sclerosis										
Femur, Medial									<0.001	59 (11)
0	379 (72)	31 (97)	44 (96)	1 (1)	55 (75)	114 (83)	118 (85)	16 (59)		
1	80 (15)	1 (3)	0 (0)	**64 (85)**	5 (7)	4 (3)	1 (1)	5 (19)		
2	11 (2)	0 (0)	0 (0)	**9 (12)**	0 (0)	0 (0)	0 (0)	2 (7)		
3	1 (0)	0 (0)	0 (0)	**1 (1)**	0 (0)	0 (0)	0 (0)	0 (0)		
Tibia, Medial									<0.001	59 (11)
0	369 (70)	31 (97)	43 (93)	1 (1)	59 (81)	103 (75)	117 (84)	15 (56)		
1	87 (16)	1 (3)	1 (2)	**61 (81)**	1 (1)	13 (9)	2 (1)	8 (30)		
2	14 (3)	0 (0)	0 (0)	**12 (16)**	0 (0)	2 (1)	0 (0)	0 (0)		
3	1 (0)	0 (0)	0 (0)	**1 (1)**	0 (0)	0 (0)	0 (0)	0 (0)		
Femur, Lateral									<0.001	59 (11)
0	438 (83)	1 (3)	44 (96)	75 (100)	60 (82)	117 (85)	119 (86)	22 (81)		

1	28 (5)	**26 (81)**	0 (0)	0 (0)	0 (0)	1 (1)	0 (0)	1 (4)		
2	4 (1)	**4 (12)**	0 (0)	0 (0)	0 (0)	0 (0)	0 (0)	0 (0)		
3	1 (0)	**1 (3)**	0 (0)	0 (0)	0 (0)	0 (0)	0 (0)	0 (0)		
Tibia, Lateral									<0.001	59 (11)
0	445 (84)	6 (19)	44 (96)	75 (100)	60 (82)	118 (86)	119 (86)	23 (85)		
1	22 (4)	**22 (69)**	0 (0)	0 (0)	0 (0)	0 (0)	0 (0)	0 (0)		
2	4 (1)	**4 (12)**	0 (0)	0 (0)	0 (0)	0 (0)	0 (0)	0 (0)		
Chondrocalcinosis										
Medial§	27 (5)	0 (0)	0 (0)	0 (0)	0 (0)	0 (0)	0 (0)	**27 (100)**	<0.001	0 (0)
Lateral§	31 (6)	1 (3)	0 (0)	0 (0)	0 (0)	1 (1)	2 (1)	**27 (100)**	<0.001	0 (0)

* Data are reported as mean ± SD or N(%). Text in bold indicate significant differentiating factors among subgroups.

† AOP group, Accelerated Osteoarthritis Progression group; SOP group, Slow Osteoarthritis Progression group

‡ KL, Kellgren and Lawrence grade

§ Since the OA phenotype has only grade 0 and 1, only data of grade 1 is presented.

Subgroups 1, 2, and 3 showed high progression rates (47%, 22% and 15%, respectively), while subgroups 4, 5, 6, and 7 showed low progression rates (15%, 10%, 3% and 7%, respectively). Subgroup 1 showed deterioration in lateral JSN (100% in lateral JSN grade 1), osteophytes (34% in lateral femur osteophyte grade 2) and subchondral sclerosis (81% in lateral femur subchondral sclerosis grade 1), while subgroup 2, initially lacked lateral subchondral sclerosis (0% in lateral femur subchondral sclerosis grade 1). Subgroup 3 showed deterioration in medial JSN (89% in lateral JSN grade 1), osteophytes (16% in medial femur osteophyte grade 3) and subchondral sclerosis (85% in medial femur subchondral sclerosis grade 1), while subgroup 4, initially lacked lateral subchondral sclerosis (7% in medial femur subchondral sclerosis grade 1). Subgroup 5 is characterized by male participants with a medial-dominant OA pattern. Subgroup 6 showed no JSN but a predominance of osteophytes regardless of location (medial/lateral, femur/tibia). Subgroup 7, the oldest group (67.8 years), has chondrocalcinosis (100% in both medial and lateral chondrocalcinosis).

Supplementary Table S5. Radiographic Phenotype Changes from Baseline to 48 Months of Subgroups in the Train Set*

	Subgroup 1 (N = 63)			Subgroup 2 (N = 75)			Subgroup 3 (N = 233)			Subgroup 4 (N = 180)			Subgroup 5 (N = 343)			Subgroup 6 (N = 308)			Subgroup 7 (N = 64)			Missing	
	Baseline	Δ	48 mo	Baseline	Δ	48 mo	Baseline	Δ	48 mo	Baseline	Δ	48 mo	Baseline	Δ	48 mo	Baseline	Δ	48 mo	Baseline	Δ	48 mo	Baseline	48 mo
KL grade†																						0 (0)	9 (1)
2	63 (100)	**-33 (-52)**	30 (48)	75 (100)	**-20 (-27)**	55 (73)	233 (100)	**-58 (-25)**	175 (75)	180 (100)	**-21 (-12)**	159 (88)	343 (100)	**-30 (-9)**	313 (91)	308 (100)	**-23 (-7)**	285 (93)	64 (100)	**-8 (-12)**	56 (88)		
3	0 (0)	**+32 (+51)**	32 (51)	0 (0)	**+19 (+25)**	19 (25)	0 (0)	**+50 (+21)**	50 (21)	0 (0)	**+19 (+11)**	19 (11)	0 (0)	**+29 (+8)**	29 (8)	0 (0)	**+18 (+6)**	18 (6)	0 (0)	**+6 (+9)**	6 (9)		
4	0 (0)	+1 (+2)	1 (2)	0 (0)	0	0 (0)	0 (0)	+6 (+3)	6 (3)	0 (0)	+1 (+1)	1 (1)	0 (0)	+1 (+0)	1 (0)	0 (0)	+1 (+0)	1 (0)	0 (0)	+1 (+2)	1 (2)		
Joint Space Narrowing																							
Medial																						0 (0)	9 (1)
0	59 (94)	-3 (-5)	56 (89)	69 (92)	**-5 (-7)**	64 (85)	26 (11)	-9 (-4)	17 (7)	0 (0)	+1 (+1)	1 (1)	158 (46)	-11 (-3)	147 (43)	308 (100)	**-26 (-8)**	282 (92)	32 (50)	**-3 (-5)**	29 (45)		
1	4 (6)	0	4 (6)	6 (8)	+2 (+3)	8 (11)	207 (89)	**-59 (-25)**	148 (64)	180 (100)	**-27 (-15)**	153 (85)	185 (54)	**-31 (-9)**	154 (45)	0 (0)	+7 (+2)	7 (2)	32 (50)	**-3 (-5)**	29 (45)		
2	0 (0)	+3 (+5)	3 (5)	0 (0)	+1 (+1)	1 (1)	0 (0)	**+42 (+18)**	42 (18)	0 (0)	**+22 (+12)**	22 (12)	0 (0)	**+32 (+9)**	32 (9)	0 (0)	+12 (+4)	12 (4)	0 (0)	**+4 (+6)**	4 (6)		
3	0 (0)	0	0 (0)	0 (0)	+1 (+1)	1 (1)	0 (0)	**+24 (+10)**	24 (10)	0 (0)	+3 (+2)	3 (2)	0 (0)	+10 (+3)	10 (3)	0 (0)	+3 (+1)	3 (1)	0 (0)	+1 (+2)	1 (2)		
Lateral																						0 (0)	9 (1)
0	4 (6)	-1 (-2)	3 (5)	0 (0)	+1 (+1)	1 (1)	229 (98)	**-6 (-3)**	223 (96)	180 (100)	-1 (-1)	179 (99)	343 (100)	-5 (-1)	338 (99)	308 (100)	-11 (-4)	297 (96)	52 (81)	**-3 (-5)**	49 (77)		
1	59 (94)	**-37 (-59)**	22 (35)	75 (100)	**-24 (-32)**	51 (68)	4 (2)	+1 (+0)	5 (2)	0 (0)	0	0 (0)	0 (0)	+3 (+1)	3 (1)	0 (0)	+3 (+1)	3 (1)	12 (19)	-2 (-3)	10 (16)		
2	0 (0)	**+22 (+35)**	22 (35)	0 (0)	**+16 (+21)**	16 (21)	0 (0)	+2 (+1)	2 (1)	0 (0)	0	0 (0)	0 (0)	+1 (+0)	1 (0)	0 (0)	+2 (+1)	2 (1)	0 (0)	**+3 (+5)**	3 (5)		
3	0 (0)	**+16 (+25)**	16 (25)	0 (0)	**+6 (+8)**	6 (8)	0 (0)	+1 (+0)	1 (0)	0 (0)	0	0 (0)	0 (0)	+1 (+0)	1 (0)	0 (0)	+2 (+1)	2 (1)	0 (0)	+1 (+2)	1 (2)		
Osteophyte																							
Femur, Medial																						0 (0)	9 (1)
0	38 (60)	**-6 (-10)**	32 (51)	46 (61)	**-7 (-9)**	39 (52)	64 (27)	-10 (-4)	54 (23)	67 (37)	**-19 (-11)**	48 (27)	261 (76)	**-20 (-6)**	241 (70)	129 (42)	**-34 (-11)**	95 (31)	34 (53)	**-3 (-5)**	31 (48)		
1	14 (22)	-2 (-3)	12 (19)	15 (20)	+1 (+1)	16 (21)	98 (42)	**-27 (-12)**	71 (30)	84 (47)	0	84 (47)	67 (20)	+10 (+3)	77 (22)	126 (41)	+4 (+1)	130 (42)	21 (33)	-1 (-2)	20 (31)		
2	5 (8)	**+4 (+6)**	9 (14)	6 (8)	+3 (+4)	9 (12)	35 (15)	**+17 (+7)**	52 (22)	24 (13)	**+10 (+6)**	34 (19)	8 (2)	+7 (+2)	15 (4)	42 (14)	+4 (+1)	46 (15)	2 (3)	+1 (+2)	3 (5)		
3	6 (10)	**+4 (+6)**	10 (16)	8 (11)	+2 (+3)	10 (13)	36 (15)	**+18 (+8)**	54 (23)	5 (3)	+8 (+4)	13 (7)	7 (2)	+3 (+1)	10 (3)	11 (4)	**+22 (+7)**	33 (11)	7 (11)	+2 (+3)	9 (14)		
Tibia, Medial																						0 (0)	9 (1)
0	31 (49)	**-4 (-6)**	27 (43)	33 (44)	**-5 (-7)**	28 (37)	21 (9)	-8 (-3)	13 (6)	37 (21)	**-11 (-6)**	26 (14)	99 (29)	**-16 (-5)**	83 (24)	109 (35)	**-28 (-9)**	81 (26)	27 (42)	**-5 (-8)**	22 (34)		
1	29 (46)	+1 (+2)	30 (48)	36 (48)	-1 (-1)	35 (47)	173 (74)	**-40 (-17)**	133 (57)	132 (73)	-7 (-4)	125 (69)	228 (66)	+3 (+1)	231 (67)	168 (55)	**+14 (+5)**	182 (59)	34 (53)	0	34 (53)		
2	1 (2)	**+3 (+5)**	4 (6)	4 (5)	+2 (+3)	6 (8)	27 (12)	**+28 (+12)**	55 (24)	11 (6)	**+12 (+7)**	23 (13)	16 (5)	+6 (+2)	22 (6)	27 (9)	+3 (+1)	30 (10)	2 (3)	**+3 (+5)**	5 (8)		
3	2 (3)	0	2 (3)	2 (3)	+3 (+4)	5 (7)	12 (5)	**+18 (+8)**	30 (13)	0 (0)	+5 (+3)	5 (3)	0 (0)	+7 (+2)	7 (2)	4 (1)	+7 (+2)	11 (4)	1 (2)	+1 (+2)	2 (3)		
Femur, Lateral																						0 (0)	9 (1)
0	20 (32)	**-4 (-6)**	16 (25)	27 (36)	**-5 (-7)**	22 (29)	147 (63)	**-16 (-7)**	131 (56)	123 (68)	**-11 (-6)**	112 (62)	190 (55)	-15 (-4)	175 (51)	149 (48)	**-20 (-6)**	129 (42)	32 (50)	**-3 (-5)**	29 (45)		
1	22 (35)	**-6 (-10)**	16 (25)	33 (44)	**-6 (-8)**	27 (36)	53 (23)	+4 (+2)	57 (24)	49 (27)	+6 (+3)	55 (31)	134 (39)	+9 (+3)	143 (42)	116 (38)	+4 (+1)	120 (39)	21 (33)	-1 (-2)	20 (31)		
2	11 (17)	**+5 (+8)**	16 (25)	8 (11)	**+7 (+9)**	15 (20)	21 (9)	+7 (+3)	28 (12)	6 (3)	+1 (+1)	7 (4)	17 (5)	+4 (+1)	21 (6)	36 (12)	+4 (+1)	40 (13)	5 (8)	0	5 (8)		
3	10 (16)	**+5 (+8)**	15 (24)	7 (9)	+3 (+4)	10 (13)	12 (5)	+3 (+1)	15 (6)	2 (1)	+3 (+2)	5 (3)	2 (1)	+2 (+1)	4 (1)	7 (2)	+8 (+3)	15 (5)	6 (9)	**+3 (+5)**	9 (14)		
Tibia, Lateral																						0 (0)	9 (1)
0	16 (25)	**-3 (-5)**	13 (21)	29 (39)	**-6 (-8)**	23 (31)	145 (62)	**-15 (-6)**	130 (56)	124 (69)	-6 (-3)	118 (66)	232 (68)	**-18 (-5)**	214 (62)	140 (45)	-12 (-4)	128 (42)	25 (39)`	-1 (-2)	24 (38)		
1	31 (49)	**-16 (-25)**	15 (24)	29 (39)	**-4 (-5)**	25 (33)	69 (30)	+3 (+1)	72 (31)	44 (24)	0	44 (24)	102 (30)	+10 (+3)	112 (33)	146 (47)	**-15 (-5)**	131 (43)	36 (56)	**-3 (-5)**	33 (52)		
2	11 (17)	**+5 (+8)**	16 (25)	9 (12)	**+4 (+5)**	13 (17)	7 (3)	+2 (+1)	9 (4)	9 (5)	-3 (-2)	6 (3)	7 (2)	+7 (+2)	14 (4)	15 (5)	**+14 (+5)**	29 (9)	2 (3)	**+3 (+5)**	5 (8)		
3	5 (8)	**+14 (+22)**	19 (30)	8 (11)	**+5 (+7)**	13 (17)	12 (5)	+8 (+3)	20 (9)	3 (2)	**+8 (+4)**	11 (6)	2 (1)	+1 (+0)	3 (1)	7 (2)	+9 (+3)	16 (5)	1 (2)	0	1 (2)		
Subchondral sclerosis																							
Femur, Medial																						111 (9)	11 (1)
0	59 (94)	**-3 (-5)**	56 (89)	63 (84)	+6 (+8)	69 (92)	2 (1)	+2 (+1)	4 (2)	153 (85)	**-9 (-5)**	144 (80)	305 (89)	+3 (+1)	308 (90)	270 (88)	+12 (+4)	282 (92)	43 (67)	+6 (+9)	49 (77)		
1	4 (6)	+1 (+2)	5 (8)	2 (3)	+2 (+3)	4 (5)	204 (88)	**-39 (-17)**	165 (71)	6 (3)	**+25 (+14)**	31 (17)	1 (0)	**+23 (+7)**	24 (7)	3 (1)	+8 (+3)	11 (4)	10 (16)	+1 (+2)	11 (17)		
2	0 (0)	+2 (+3)	2 (3)	0 (0)	+1 (+1)	1 (1)	27 (12)	**+31 (+13)**	58 (25)	0 (0)	+4 (+2)	4 (2)	1 (0)	+10 (+3)	11 (3)	0 (0)	+9 (+3)	9 (3)	2 (3)	+1 (+2)	3 (5)		
3	0 (0)	0	0 (0)	0 (0)	0	0 (0)	0 (0)	+4 (+2)	4 (2)	0 (0)	0	0 (0)	0 (0)	0	0 (0)	0 (0)	0	0 (0)	0 (0)	0	0 (0)		
Tibia, Medial																						111 (9)	11 (1)
0	57 (90)	-2 (-3)	55 (87)	63 (84)	+2 (+3)	65 (87)	12 (5)	-5 (-2)	7 (3)	157 (87)	**-15 (-8)**	142 (79)	277 (81)	+2 (+1)	279 (81)	270 (88)	+10 (+3)	280 (91)	44 (69)	+5 (+8)	49 (77)		
1	6 (10)	-1 (-2)	5 (8)	2 (3)	**+5 (+7)**	7 (9)	193 (83)	**-28 (-12)**	165 (71)	2 (1)	**+28 (+16)**	30 (17)	29 (8)	**+22 (+6)**	51 (15)	3 (1)	+12 (+4)	15 (5)	10 (16)	+2 (+3)	12 (19)		
2	0 (0)	**+3 (+5)**	3 (5)	0 (0)	+2 (+3)	2 (3)	28 (12)	**+27 (+12)**	55 (24)	0 (0)	+6 (+3)	6 (3)	1 (0)	+11 (+3)	12 (3)	0 (0)	+7 (+2)	7 (2)	1 (2)	+1 (+2)	2 (3)		
3	0 (0)	0	0 (0)	0 (0)	0	0 (0)	0 (0)	+4 (+2)	4 (2)	0 (0)	+1 (+1)	1 (1)	0 (0)	+1 (+0)	1 (0)	0 (0)	0	0 (0)	0 (0)	0	0 (0)		
Femur, Lateral																						111 (9)	11 (1)
0	5 (8)	-2 (-3)	3 (5)	65 (87)	**-5 (-7)**	60 (80)	230 (99)	-5 (-2)	225 (97)	159 (88)	+20 (+11)	179 (99)	307 (90)	+35 (+10)	342 (100)	273 (89)	+27 (+9)	300 (97)	51 (80)	+7 (+11)	58 (91)		
1	54 (86)	**-10 (-16)**	44 (70)	0 (0)	**+10 (+13)**	10 (13)	3 (1)	+2 (+1)	5 (2)	0 (0)	0	0 (0)	0 (0)	+1 (+0)	1 (0)	0 (0)	0	0 (0)	4 (6)	0	4 (6)		
2	4 (6)	**+12 (+19)**	16 (25)	0 (0)	**+4 (+5)**	4 (5)	0 (0)	+1 (+0)	1 (0)	0 (0)	0	0 (0)	0 (0)	0	0 (0)	0 (0)	+2 (+1)	2 (1)	0 (0)	+1 (+2)	1 (2)		

Tibia, Lateral																						111 (9)	11 (1)
0	14 (22)	-1 (-2)	13 (21)	65 (87)	-3 (-4)	62 (83)	232 (100)	-4 (-2)	228 (98)	159 (88)	+20 (+11)	179 (99)	307 (90)	+33 (+10)	340 (99)	273 (89)	+27 (+9)	300 (97)	54 (84)	+5 (+8)	59 (92)		
1	42 (67)	**-11 (-17)**	31 (49)	0 (0)	**+7 (+9)**	7 (9)	1 (0)	+2 (+1)	3 (1)	0 (0)	0	0 (0)	0 (0)	+2 (+1)	2 (1)	0 (0)	0	0 (0)	1 (2)	+2 (+3)	3 (5)		
2	7 (11)	**+12 (+19)**	19 (30)	0 (0)	**+5 (+7)**	5 (7)	0 (0)	0	0 (0)	0 (0)	0	0 (0)	0 (0)	+1 (+0)	1 (0)	0 (0)	+2 (+1)	2 (1)	0 (0)	+1 (+2)	1 (2)		
Chondrocalcinosis																							
Medial‡	0 (0)	+1 (+2)	1 (2)	0 (0)	0	0 (0)	1 (0)	0	1 (0)	0 (0)	+3 (+2)	3 (2)	1 (0)	+3 (+1)	4 (1)	0 (0)	+3 (+1)	3 (1)	64 (100)	-1 (-2)	63 (98)	1 (0)	11 (1)
Lateral‡	0 (0)	0	0 (0)	0 (0)	+3 (+4)	3 (4)	2 (1)	+2 (+1)	4 (2)	0 (0)	+2 (+1)	2 (1)	2 (1)	+1 (+0)	3 (1)	0 (0)	+3 (+1)	3 (1)	64 (100)	-1 (-2)	63 (98)	1 (0)	11 (1)

*Data are reported as N(%). Δ (delta) indicates difference of N(%) from baseline to 48 month (48 mo) in each phenotype grade. Values exhibiting a change of more than 5% across subgroup sample sizes are highlighted in bold. The increase in grade 0 is not indicative of the disappearance of the specific phenotype but a reduction in missing values. Therefore, increased percentage of grade 0 more than 5% is not emphasized in bold.

† KL grade, Kellgren and Lawrence grade

‡ Since the OA phenotype has only grade 0 and 1, only data of grade 1 is presented.

Supplementary Table S6. Radiographic Phenotype Changes from Baseline to 48 Months of Subgroups in the Test Set*

	Subgroup 1 (N = 32)			Subgroup 2 (N = 46)			Subgroup 3 (N = 75)			Subgroup 4 (N = 73)			Subgroup 5 (N = 138)			Subgroup 6 (N = 139)			Subgroup 7 (N = 27)			Missing	
	Baseline	Δ	48 mo	Baseline	Δ	48 mo	Baseline	Δ	48 mo	Baseline	Δ	48 mo	Baseline	Δ	48 mo	Baseline	Δ	48 mo	Baseline	Δ	48 mo	Baseline	48 mo
KL grade†																						0 (0)	8 (2)
2	32 (100)	**-16 (-50)**	16 (50)	46 (100)	**-11 (-24)**	35 (76)	75 (100)	**-12 (-16)**	63 (84)	73 (100)	**-12 (-16)**	61 (84)	138 (100)	**-14 (-10)**	124 (90)	139 (100)	-6 (-4)	133 (96)	27 (100)	**-4 (-15)**	23 (85)		
3	0 (0)	**+13 (+41)**	13 (41)	0 (0)	**+9 (+20)**	9 (20)	0 (0)	**+11 (+15)**	11 (15)	0 (0)	**+10 (+14)**	10 (14)	0 (0)	**+13 (+9)**	13 (9)	0 (0)	+4 (+3)	4 (3)	0 (0)	**+2 (+7)**	2 (7)		
4	0 (0)	**+2 (+6)**	2 (6)	0 (0)	+1 (+2)	1 (2)	0 (0)	0	0 (0)	0 (0)	+1 (+1)	1 (1)	0 (0)	+1 (+1)	1 (1)	0 (0)	0	0 (0)	0 (0)	0	0 (0)		
Joint Space Narrowing																							
Medial																						0 (0)	8 (2)
0	31 (97)	-1 (-3)	30 (94)	40 (87)	**-4 (-9)**	36 (78)	8 (11)	+1 (+1)	9 (12)	0 (0)	0	0 (0)	50 (36)	-4 (-3)	46 (33)	139 (100)	**-8 (-6)**	131 (94)	11 (41)	-1 (-4)	10 (37)		
1	1 (3)	0	1 (3)	6 (13)	0	6 (13)	67 (89)	**-15 (-20)**	52 (69)	73 (100)	**-12 (-16)**	61 (84)	88 (64)	**-10 (-7)**	78 (57)	0 (0)	+3 (+2)	3 (2)	16 (59)	**-2 (-7)**	14 (52)		
2	0 (0)	0	0 (0)	0 (0)	**+3 (+7)**	3 (7)	0 (0)	**+11 (+15)**	11 (15)	0 (0)	**+9 (+12)**	9 (12)	0 (0)	**+11 (+8)**	11 (8)	0 (0)	+1 (+1)	1 (1)	0 (0)	0	0 (0)		
3	0 (0)	0	0 (0)	0 (0)	0	0 (0)	0 (0)	+2 (+3)	2 (3)	0 (0)	+2 (+3)	2 (3)	0 (0)	+3 (+2)	3 (2)	0 (0)	+2 (+1)	2 (1)	0 (0)	+1 (+4)	1 (4)		
Lateral																						0 (0)	8 (2)
0	0 (0)	0	0 (0)	0 (0)	0	0 (0)	75 (100)	-4 (-5)	71 (95)	73 (100)	-1 (-1)	72 (99)	138 (100)	-2 (-1)	136 (99)	139 (100)	**-8 (-6)**	131 (94)	23 (85)	**-2 (-7)**	21 (78)		
1	32 (100)	**-18 (-56)**	14 (44)	46 (100)	**-11 (-24)**	35 (76)	0 (0)	+2 (+3)	2 (3)	0 (0)	0	0 (0)	0 (0)	+1 (+1)	1 (1)	0 (0)	+4 (+3)	4 (3)	4 (15)	-1 (-4)	3 (11)		
2	0 (0)	**+10 (+31)**	10 (31)	0 (0)	**+7 (+15)**	7 (15)	0 (0)	+1 (+1)	1 (1)	0 (0)	0	0 (0)	0 (0)	+1 (+1)	1 (1)	0 (0)	+2 (+1)	2 (1)	0 (0)	0	0 (0)		
3	0 (0)	**+7 (+22)**	7 (22)	0 (0)	**+3 (+7)**	3 (7)	0 (0)	0	0 (0)	0 (0)	0	0 (0)	0 (0)	0	0 (0)	0 (0)	0	0 (0)	0 (0)	+1 (+4)	1 (4)		
Osteophyte																							
Femur, Medial																						0 (0)	8 (2)
0	24 (75)	**-2 (-6)**	22 (69)	26 (57)	**-5 (-11)**	21 (46)	18 (24)	**-7 (-9)**	11 (15)	27 (37)	**-6 (-8)**	21 (29)	96 (70)	**-10 (-7)**	86 (62)	54 (39)	**-12 (-9)**	42 (30)	16 (59)	**-2 (-7)**	14 (52)		
1	4 (12)	0	4 (12)	10 (22)	**+5 (+11)**	15 (33)	32 (43)	-2 (-3)	30 (40)	28 (38)	-1 (-1)	27 (37)	32 (23)	**+8 (+6)**	40 (29)	56 (40)	+1 (+1)	57 (41)	7 (26)	-1 (-4)	6 (22)		
2	1 (3)	+1 (+3)	2 (6)	6 (13)	-1 (-2)	5 (11)	13 (17)	+3 (+4)	16 (21)	9 (12)	0	9 (12)	9 (7)	0	9 (7)	23 (17)	+1 (+1)	24 (17)	3 (11)	+1 (+4)	4 (15)		
3	3 (9)	0	3 (9)	4 (9)	0	4 (9)	12 (16)	**+5 (+7)**	17 (23)	9 (12)	**+6 (+8)**	15 (21)	1 (1)	+2 (+1)	3 (2)	6 (4)	**+8 (+6)**	14 (10)	1 (4)	0	1 (4)		
Tibia, Medial																						0 (0)	8 (2)
0	17 (53)	+1 (+3)	18 (56)	22 (48)	**-5 (-11)**	17 (37)	9 (12)	-3 (-4)	6 (8)	19 (26)	-3 (-4)	16 (22)	36 (26)	-6 (-4)	30 (22)	54 (39)	**-14 (-10)**	40 (29)	4 (15)	**-2 (-7)**	2 (7)		
1	13 (41)	-1 (-3)	12 (38)	22 (48)	**+3 (+7)**	25 (54)	46 (61)	**-8 (-11)**	38 (51)	49 (67)	-4 (-5)	45 (62)	93 (67)	0	93 (67)	72 (52)	+6 (+4)	78 (56)	22 (81)	0	22 (81)		
2	2 (6)	-1 (-3)	1 (3)	2 (4)	0	2 (4)	18 (24)	+3 (+4)	21 (28)	4 (5)	+4 (+5)	8 (11)	9 (7)	+3 (+2)	12 (9)	11 (8)	+3 (+2)	14 (10)	1 (4)	0	1 (4)		
3	0 (0)	0	0 (0)	0 (0)	+1 (+2)	1 (2)	2 (3)	**+7 (+9)**	9 (12)	1 (1)	+2 (+3)	3 (4)	0 (0)	+3 (+2)	3 (2)	2 (1)	+3 (+2)	5 (4)	0 (0)	0	0 (0)		
Femur, Lateral																						0 (0)	8 (2)
0	6 (19)	-1 (-3)	5 (16)	23 (50)	**-8 (-17)**	15 (33)	43 (57)	**-6 (-8)**	37 (49)	40 (55)	**-7 (-10)**	33 (45)	76 (55)	0	76 (55)	63 (45)	**-12 (-9)**	51 (37)	16 (59)	**-2 (-7)**	14 (52)		
1	14 (44)	**-4 (-12)**	10 (31)	13 (28)	**+3 (+7)**	16 (35)	20 (27)	+4 (+5)	24 (32)	27 (37)	**+5 (+7)**	32 (44)	54 (39)	-1 (-1)	53 (38)	60 (43)	+1 (+1)	61 (44)	7 (26)	-1 (-4)	6 (22)		
2	11 (34)	**-2 (-6)**	9 (28)	8 (17)	**+3 (+7)**	11 (24)	8 (11)	-2 (-3)	6 (8)	6 (8)	-1 (-1)	5 (7)	7 (5)	+1 (+1)	8 (6)	11 (8)	**+8 (+6)**	19 (14)	3 (11)	+1 (+4)	4 (15)		
3	1 (3)	**+6 (+19)**	7 (22)	2 (4)	+1 (+2)	3 (7)	4 (5)	+3 (+4)	7 (9)	0 (0)	+2 (+3)	2 (3)	1 (1)	0	1 (1)	5 (4)	+1 (+1)	6 (4)	1 (4)	0	1 (4)		
Tibia, Lateral																						0 (0)	8 (2)
0	6 (19)	**-2 (-6)**	4 (12)	16 (35)	-1 (-2)	15 (33)	51 (68)	-2 (-3)	49 (65)	42 (58)	**-6 (-8)**	36 (49)	92 (67)	-6 (-4)	86 (62)	63 (45)	**-8 (-6)**	55 (40)	13 (48)	+1 (+4)	14 (52)		
1	17 (53)	**-4 (-12)**	13 (41)	25 (54)	**-5 (-11)**	20 (43)	19 (25)	0	19 (25)	27 (37)	+1 (+1)	28 (38)	40 (29)	+5 (+4)	45 (33)	58 (42)	-3 (-2)	55 (40)	8 (30)	**-4 (-15)**	4 (15)		
2	6 (19)	-1 (-3)	5 (16)	3 (7)	**+3 (+7)**	6 (13)	5 (7)	-2 (-3)	3 (4)	3 (4)	+2 (+3)	5 (7)	2 (1)	+1 (+1)	3 (2)	11 (8)	+4 (+3)	15 (11)	4 (15)	**-2 (-7)**	2 (7)		
3	3 (9)	**+6 (+19)**	9 (28)	2 (4)	+2 (+4)	4 (9)	0 (0)	+3 (+4)	3 (4)	1 (1)	+2 (+3)	3 (4)	4 (3)	0	4 (3)	7 (5)	+5 (+4)	12 (9)	2 (7)	**+3 (+11)**	5 (19)		
Subchondral sclerosis																							
Femur, Medial																						59 (11)	8 (2)
0	31 (97)	-1 (-3)	30 (94)	44 (96)	0	44 (96)	1 (1)	+1 (+1)	2 (3)	55 (75)	+1 (+1)	56 (77)	114 (83)	+5 (+4)	119 (86)	118 (85)	+14 (+10)	132 (95)	16 (59)	+1 (+4)	17 (63)		
1	1 (3)	0	1 (3)	0 (0)	+1 (+2)	1 (2)	64 (85)	-3 (-4)	61 (81)	5 (7)	**+6 (+8)**	11 (15)	4 (3)	**+11 (+8)**	15 (11)	1 (1)	0	1 (1)	5 (19)	0	5 (19)		
2	0 (0)	0	0 (0)	0 (0)	0	0 (0)	9 (12)	+1 (+1)	10 (13)	0 (0)	+4 (+5)	4 (5)	0 (0)	+4 (+3)	4 (3)	0 (0)	+4 (+3)	4 (3)	2 (7)	+1 (+4)	3 (11)		
3	0 (0)	0	0 (0)	0 (0)	0	0 (0)	1 (1)	0	1 (1)	0 (0)	+1 (+1)	1 (1)	0 (0)	0	0 (0)	0 (0)	0	0 (0)	0 (0)	0	0 (0)		
Tibia, Medial																						59 (11)	8 (2)
0	31 (97)	-1 (-3)	30 (94)	43 (93)	-1 (-2)	42 (91)	1 (1)	+1 (+1)	2 (3)	59 (81)	-4 (-5)	55 (75)	103 (75)	+6 (+4)	109 (79)	117 (84)	+14 (+10)	131 (94)	15 (56)	0	15 (56)		
1	1 (3)	0	1 (3)	1 (2)	+1 (+2)	2 (4)	61 (81)	-3 (-4)	58 (77)	1 (1)	**+10 (+14)**	11 (15)	13 (9)	+7 (+5)	20 (14)	2 (1)	+1 (+1)	3 (2)	8 (30)	+1 (+4)	9 (33)		
2	0 (0)	0	0 (0)	0 (0)	+1 (+2)	1 (2)	12 (16)	+1 (+1)	13 (17)	0 (0)	+3 (+4)	3 (4)	2 (1)	+7 (+5)	9 (7)	0 (0)	+3 (+2)	3 (2)	0 (0)	+1 (+4)	1 (4)		
3	0 (0)	0	0 (0)	0 (0)	0	0 (0)	1 (1)	0	1 (1)	0 (0)	+3 (+4)	3 (4)	0 (0)	0	0 (0)	0 (0)	0	0 (0)	0 (0)	0	0 (0)		
Femur, Lateral																						59 (11)	8 (2)

0	1 (3)	-1 (-3)	0 (0)	44 (96)	**-13 (-28)**	31 (67)	75 (100)	-2 (-3)	73 (97)	60 (82)	+12 (+16)	72 (99)	117 (85)	+20 (+14)	137 (99)	119 (86)	+17 (+12)	136 (98)	22 (81)	0	22 (81)		
1	26 (81)	**-6 (-19)**	20 (62)	0 (0)	**+13 (+28)**	13 (28)	0 (0)	+1 (+1)	1 (1)	0 (0)	0	0 (0)	1 (1)	0	1 (1)	0 (0)	+1 (+1)	1 (1)	1 (4)	**+2 (+7)**	3 (11)		
2	4 (12)	**+5 (+16)**	9 (28)	0 (0)	+1 (+2)	1 (2)	0 (0)	0	0 (0)	0 (0)	0	0 (0)	0 (0)	0	0 (0)	0 (0)	0	0 (0)	0 (0)	0	0 (0)		
3	1 (3)	+1 (+3)	2 (6)	0 (0)	0	0 (0)	0 (0)	0	0 (0)	0 (0)	0	0 (0)	0 (0)	0	0 (0)	0 (0)	0	0 (0)	0 (0)	0	0 (0)		
Tibia, Lateral																						59 (11)	8 (2)
0	6 (19)	**-4 (-12)**	2 (6)	44 (96)	**-8 (-17)**	36 (78)	75 (100)	-1 (-1)	74 (99)	60 (82)	+11 (+15)	71 (97)	118 (86)	+20 (+14)	138 (100)	119 (86)	+17 (+12)	136 (98)	23 (85)	+1 (+4)	24 (89)		
1	22 (69)	**-3 (-9)**	19 (59)	0 (0)	**+7 (+15)**	7 (15)	0 (0)	0	0 (0)	0 (0)	+1 (+1)	1 (1)	0 (0)	0	0 (0)	0 (0)	+1 (+1)	1 (1)	0 (0)	+1 (+4)	1 (4)		
2	4 (12)	**+4 (+12)**	8 (25)	0 (0)	+2 (+4)	2 (4)	0 (0)	0	0 (0)	0 (0)	0	0 (0)	0 (0)	0	0 (0)	0 (0)	0	0 (0)	0 (0)	0	0 (0)		
3	0 (0)	**+2 (+6)**	2 (6)	0 (0)	0	0 (0)	0 (0)	0	0 (0)	0 (0)	0	0 (0)	0 (0)	0	0 (0)	0 (0)	0	0 (0)	0 (0)	0	0 (0)		
Chondrocalcinosis																							
Medial‡	0 (0)	**+3 (+9)**	3 (9)	0 (0)	0	0 (0)	0 (0)	0	0 (0)	0 (0)	+1 (+1)	1 (1)	0 (0)	+2 (+1)	2 (1)	0 (0)	+4 (+3)	4 (3)	27 (100)	**-3 (-11)**	24 (89)		
Lateral‡	1 (3)	+1 (+3)	2 (6)	0 (0)	0	0 (0)	0 (0)	+1 (+1)	1 (1)	0 (0)	0	0 (0)	1 (1)	+2 (+1)	3 (2)	2 (1)	+4 (+3)	6 (4)	27 (100)	**-3 (-11)**	24 (89)		

*Data are reported as N(%). Δ (delta) indicates difference of N(%) from baseline to 48 month (48 mo) in each phenotype grade. Values exhibiting a change of more than 5% across subgroup sample sizes are highlighted in bold. The increase in grade 0 is not indicative of the disappearance of the specific phenotype but a reduction in missing values. Therefore, increased percentage of grade 0 more than 5% is not emphasized in bold.

† KL grade, Kellgren and Lawrence grade

‡ Since the OA phenotype has only grade 0 and 1, only data of grade 1 is presented.

Supplementary Table S7. Percentage of Knee Replacement or Progression to KL grade 4 (Survivals) of Each Subgroup for Train and Test Set

Training Set					
	1 y	3 y	5 y	7 y	9 y
Subgroup 1 (n = 63)	1.000	1.000	0.984	0.933	0.844
Subgroup 2 (n = 75)	1.000	0.987	0.987	0.928	0.912
Subgroup 3 (n = 233)	1.000	0.991	0.982	0.946	0.916
Subgroup 4 (n = 180)	0.994	0.994	0.994	0.983	0.976
Subgroup 5 (n = 343)	1.000	1.000	0.994	0.981	0.961
Subgroup 6 (n = 308)	1.000	0.997	0.980	0.963	0.949
Subgroup 7 (n = 64)	1.000	1.000	0.953	0.902	0.902
Test Set					
	1 y	3 y	5 y	7 y	9 y
Subgroup 1 (n = 32)	1.000	0.969	0.936	0.936	0.795
Subgroup 2 (n = 46)	1.000	1.000	0.956	0.956	0.931
Subgroup 3 (n = 75)	1.000	1.000	0.973	0.973	0.958
Subgroup 4 (n = 73)	1.000	0.986	0.986	0.986	0.970
Subgroup 5 (n = 138)	1.000	1.000	0.985	0.978	0.939
Subgroup 6 (n = 139)	1.000	0.986	0.986	0.978	0.978
Subgroup 7 (n = 27)	1.000	0.963	0.926	0.886	0.886

STROBE Statement—Checklist of items that should be included in reports of ***cohort studies***

	Item No	Recommendation	Page No
**Title and abstract**	1	(*a*) Indicate the study's design with a commonly used term in the title or the abstract	1
		(*b*) Provide in the abstract an informative and balanced summary of what was done and what was found	1–2
**Introduction**			
Background/rationale	2	Explain the scientific background and rationale for the investigation being reported	2–3
Objectives	3	State specific objectives, including any prespecified hypotheses	3
**Methods**			
Study design	4	Present key elements of study design early in the paper	4
Setting	5	Describe the setting, locations, and relevant dates, including periods of recruitment, exposure, follow-up, and data collection	4
Participants	6	(*a*) Give the eligibility criteria, and the sources and methods of selection of participants. Describe methods of follow-up	4
		(*b*) For matched studies, give matching criteria and number of exposed and unexposed	
Variables	7	Clearly define all outcomes, exposures, predictors, potential confounders, and effect modifiers. Give diagnostic criteria, if applicable	4–6
Data sources/ measurement	8*	For each variable of interest, give sources of data and details of methods of assessment (measurement). Describe comparability of assessment methods if there is more than one group	4–6
Bias	9	Describe any efforts to address potential sources of bias	15–16
Study size	10	Explain how the study size was arrived at	4
Quantitative variables	11	Explain how quantitative variables were handled in the analyses. If applicable, describe which groupings were chosen and why	5–8
Statistical methods	12	(*a*) Describe all statistical methods, including those used to control for confounding	7–8
		(*b*) Describe any methods used to examine subgroups and interactions	6–8
		(*c*) Explain how missing data were addressed	5, 15
		(*d*) If applicable, explain how loss to follow-up was addressed	
		(*e*) Describe any sensitivity analyses	7–8
**Results**			
Participants	13*	(a) Report numbers of individuals at each stage of study—eg numbers potentially eligible, examined for eligibility, confirmed eligible, included in the study, completing follow-up, and analysed	9
		(b) Give reasons for non-participation at each stage	9
		(c) Consider use of a flow diagram	20
Descriptive data	14*	(a) Give characteristics of study participants (eg demographic, clinical, social) and information on exposures and potential confounders	9
		(b) Indicate number of participants with missing data for each variable of interest	5
		(c) Summarise follow-up time (eg, average and total amount)	9
Outcome data	15*	Report numbers of outcome events or summary measures over time	9–12

Main results	16	(*a*) Give unadjusted estimates and, if applicable, confounder-adjusted estimates and their precision (eg, 95% confidence interval). Make clear which confounders were adjusted for and why they were included	9–12
		(*b*) Report category boundaries when continuous variables were categorized	5–6
		(*c*) If relevant, consider translating estimates of relative risk into absolute risk for a meaningful time period	
Other analyses	17	Report other analyses done—eg analyses of subgroups and interactions, and sensitivity analyses	10–12
**Discussion**			
Key results	18	Summarise key results with reference to study objectives	12–13
Limitations	19	Discuss limitations of the study, taking into account sources of potential bias or imprecision. Discuss both direction and magnitude of any potential bias	14–16
Interpretation	20	Give a cautious overall interpretation of results considering objectives, limitations, multiplicity of analyses, results from similar studies, and other relevant evidence	12–16
Generalisability	21	Discuss the generalisability (external validity) of the study results	16
**Other information**			
Funding	22	Give the source of funding and the role of the funders for the present study and, if applicable, for the original study on which the present article is based	17

*Give information separately for exposed and unexposed groups.

Note: An Explanation and Elaboration article discusses each checklist item and gives methodological background and published examples of transparent reporting. The STROBE checklist is best used in conjunction with this article (freely available on the Web sites of PLoS Medicine at http://www.plosmedicine.org/, Annals of Internal Medicine at http://www.annals.org/, and Epidemiology at http://www.epidem.com/). Information on the STROBE Initiative is available at http://www.strobe-statement.org.
